# Time for Quiescence: Modelling Quiescent Behaviour in Testing via Time-Outs in Timed Automata

**DOI:** 10.1007/s42979-026-05277-x

**Published:** 2026-11-06

**Authors:** Laura Brandán Briones, Marcus Gerhold, Petra van den Bos, Mariëlle Stoelinga

**Affiliations:** 1 FaMAF, Universidad Nacional de Córdoba, Córdoba, Argentina; 2 Formal Methods and Tools, University of Twente, Enschede, The Netherlands; 3 Department of Software Science, Radboud University, Nijmegen, The Netherlands

**Keywords:** Model-based testing, Timed automata, Quiescence, Conformance testing

## Abstract

Model-based testing (MBT) derives test suites from a behavioural specification of the system under test. In industrial practice, engineers favor simple models such as labelled transition systems (LTSs). However, handling *quiescence* (i.e., the absence of observable outputs) requires introducing a time-out to determine when quiescence can be concluded. While timed versions of model-based testing address this, they usually rely on the full expressive power of timed automata (TA), which is often more complex than needed. We introduce a lightweight lifting operator $\chi ^{\scriptstyle M}\!$ that augments LTSs with time without the overhead of general TA. Given an LTS and a user-defined timeout $\mathbf{M}>0$, $\chi ^{\scriptstyle M}\!$ adds a single clock that restricts observable outputs to occur strictly before ***M*** and makes quiescence observable exactly at time ***M***. Our contributions are as follows: (1) We show that lifting preserves input–output conformance: $\mathbf{ioco}$ coincides with timed $\mathbf{tioco}_{\mathbf{M}}$ after lifting, even in the presence of internal (“*τ *”) transitions; (2) We show that $\chi ^{\scriptstyle M}\!$ commutes with test generation: applying the lifting before or after standard **ioco** test generation yields the same test suite; (3) We show that lifted and untimed test suites assign identical verdicts to all implementations; (4) We characterise the timed languages induced by $\chi ^{\scriptstyle M}\!$ as ***M***-urgent, and ensure our construction is robust under action hiding.

## Introduction

**Quiescence in testing.** A key challenge in software testing, and in particular in model-based testing, is the treatment of *quiescence*, i.e. states where the system under test (SUT) produces no observable output. The absence of output can be meaningful behaviour: for instance, a system may be allowed to remain silent while waiting for further input, or it may indicate an error. Correctly interpreting such situations is therefore essential for assigning sound test verdicts. In practice, quiescence is typically detected by means of a time-out: if no output is observed within a fixed time bound, the system is considered quiescent.

**Model-based testing.** Model-based testing (MBT) is an effective approach to testing, as it supports automated test generation, execution, and evaluation. Central to MBT is a system specification model ${\mathbf{\mathcal {A}}}_S$ that precisely defines the intended input–output behaviour of the system. Test cases are derived automatically from ${\mathbf{\mathcal {A}}}_S$ and executed against the system under test (SUT). A test verdict (*pass* or *fail*) is then assigned: if the observed behaviour of the SUT conforms to ${\mathbf{\mathcal {A}}}_S$, the verdict is *pass*; otherwise, the verdict is *fail*.

**The input–output conformance framework.** The mathematical foundation of MBT is provided by formal conformance relations that define when an implementation correctly realises its specification. The de facto standard in this area is the *input–output conformance* (**ioco**) theory [1], which is widely used in both academic research and industrial practice. The ${\mathbf {ioco}}$ framework establishes, with mathematical precision, when an implementation model ${\mathbf{\mathcal {A}}}_I$ conforms to a specification model ${\mathbf{\mathcal {A}}}_S$, where both are given as labelled transition systems.

**Ioco**-theory provides strong correctness guarantees for model-based testing, including *soundness* (all emitted test verdicts are correct) and *completeness* (all non-conformant behaviours can in principle be detected). Under suitable assumptions, the ${\mathbf {ioco}}$ relation precisely characterises correct implementation behaviour.

**Quiescence in ioco and timed testing.** Within **ioco**-theory, quiescence is treated explicitly by extending observable traces with a special action *δ *, representing the absence of output. This is formalised via *suspension traces*, which record *δ * whenever a state is reached that has no outgoing output transitions. In this way, the observation semantics of the specification is augmented to make quiescent behaviour explicit.

In industrial practice, however, quiescence is almost always detected using time-outs: if no output is observed within a fixed bound *M*, the system is deemed quiescent. Such time-outs can be naturally modelled using timed automata, and several timed extensions of **ioco** have been proposed [2–4].

**Approach.** In this paper, we establish a precise connection between untimed **ioco** and the timed conformance relation $\mathbf{tioco}_M$. We show that modelling quiescence explicitly as a time-out preserves conformance. More concretely, we define a transformation $\chi ^{\scriptstyle M}\!$ that maps a labelled transition system ${\mathbf{\mathcal {A}}}$ to a timed automaton $\chi ^{\scriptstyle M}\!({\mathcal {A}})$, which enforces that all outputs occur strictly before a fixed time bound *M*, and that quiescence is observed exactly at time *M*. Unlike our earlier work [5], the presented setting explicitly supports internal (“*τ *”) transitions, which are observationally distinct from quiescence and therefore require careful treatment when time-outs are introduced. We prove that conformance is preserved under this transformation: if an implementation ${\mathbf{\mathcal {A}}}_I$ conforms to a specification ${\mathbf{\mathcal {A}}}_S$ in the untimed sense (i.e. ${\mathbf{\mathcal {A}}}_I\ {\mathbf {ioco}}\ {\mathbf{\mathcal {A}}}_S$), then their timed counterparts also conform (i.e. $\chi ^{\scriptstyle M}\!({\mathbf{\mathcal {A}}}_I)\ {\mathbf {tioco}} _M\ \chi ^{\scriptstyle M}\!({\mathbf{\mathcal {A}}}_S)$), for any *M>0*.

Furthermore, we show that this correspondence extends to the level of test cases. Since test cases are themselves labelled transition systems, the transformation $\chi ^{\scriptstyle M}\!$ can be applied to them directly, yielding timed test cases whose execution against $\chi ^{\scriptstyle M}\!({\mathbf{\mathcal {A}}}_I)$ produces the same verdicts as the untimed tests against ${\mathbf{\mathcal {A}}}_I$. In addition, we show that $\chi ^{\scriptstyle M}\!$ commutes with test generation: applying the transformation before or after the standard **ioco** test generation algorithm yields the same set of tests. Lastly, the newly added explicit support for internal (*τ *) transitions enables the introduction of an *action hiding operator*. We extend our previous work by analysing the impact of hiding on the observable timed language induced by the lifting operator $\chi ^{\scriptstyle M}\!$.

**Contributions.** In summary, our contributions are as follows:

- We introduce a canonic transformation $\chi ^{\scriptstyle M}\!$ that lifts untimed LTS/IOTS specifications to timed automata by adding a single clock and an explicit quiescence timeout, while preserving observable behaviour.
- We prove semantic equivalence between untimed input–output conformance (**ioco**) and timed conformance ($\mathbf{tioco}_M$) under $\chi ^{\scriptstyle M}\!$, for any timeout bound *M>0*.
- We establish a one-to-one correspondence between untimed and timed test suites, showing that test generation commutes with $\chi ^{\scriptstyle M}\!$ and that pass/fail verdicts are preserved.
- We characterise the transformed timed models as *M*-urgent and analyse the impact of action hiding on *M*-urgency.

This paper extends earlier work [5] to also include internal *τ *-transitions. These *τ *-transitions are intricate to handle in combination with quiescence because they fundamentally blur the distinction between “nothing happens” and “something happens internally”. Notably, quiescence is an observational concept, while *τ *-transitions are not. Additionally, we include the proofs of all lemmas and theorems in this paper.

**Paper overview.** Labelled Transition Systems and ioco recalls prerequisites on labelled transition systems and **ioco**, and Timed Automata and tioco the necessary theory of timed automata and $\mathbf{tioco}_M$. In Transformations we define the transformation rules from LTS into TA and prove preservation of conformance from **ioco** to $\mathbf{tioco}_M$. In Testing with TAs, we define test cases for LTS and TA, and prove commutativity of $\chi ^{\scriptstyle M}\!$ when applied on LTS and all its test cases, and that an implementation that fails a test case in the LTS setting will also do so in the TA setting. In *M*-Urgent Traces and Action Hiding we ellaborate on *M*-urgency and the relation to action hiding. Lastly, we discuss related work in Related Work and conclude the paper in Conclusion .

## Labelled Transition Systems and ioco

**Labelled transition systems (LTSs)**. In model-based testing, LTSs specify the required behaviour of a System Under Test (SUT). It is common to distinguish between input and output labels of the system explicitly: Input labels denote actions provided to the SUT, while output labels denote output actions performed by the SUT. Additionally, the model contains an internal, unobservable *τ * action, which does not belong to the sets of input or output labels.

### Definition 1

(*Labelled Transition System*) A labelled transition system (LTS) is a 4-tuple ${\mathcal {A}}= \langle S, \textit{Act}_\tau , \rightarrow , s_0\rangle$, where *S* is a finite set of states with a unique starting state $s_0\in S$, $\textit{Act}_\tau$ is the finite set of actions partitioned into input and output actions and the *τ *-action, i.e. $\textit{Act}_\tau = \textit{Act}\cup \{\tau \}$, $\textit{Act}= \textit{Act}_I\cup \textit{Act}_O$, $\textit{Act}_I\cap \textit{Act}_O= \emptyset$, and $\rightarrow \ \subseteq S\times \textit{Act}_\tau \times S$ is the transition relation.

- We write $s\xrightarrow {a}s'$ for , and $s\xrightarrow {a}$ if $s\xrightarrow {a}s'$ for some *s'∈ S* and $s\mathop{\not\rightarrow}\limits^a$ if no such *s'* exists
- If $\sigma = a_1\ldots a_n$ for $a_1,\ldots ,a_n\in \textit{Act}_\tau$, we write $s\xrightarrow {\sigma } s'$ if there are states $s_1, \ldots , s_{n-1}\in S$ such that $s\xrightarrow {a_1} s_1 \ldots s_{n-1}\xrightarrow {a_n} s'$
- We write $s\mathop {\Rightarrow }\limits ^{\epsilon }s'$ if either *s= s'* or $s\xrightarrow {\tau \ldots \tau }s'$. We write $s\mathop {\Rightarrow }\limits ^{a}s'$ for $a\in \textit{Act}$ if there are $s_1,s_2\in S$ such that $s\mathop {\Rightarrow }\limits ^{\epsilon }s_1\xrightarrow {a}s_2\mathop {\Rightarrow }\limits ^{\epsilon }s'$, and we write $s\mathop{\not\Rightarrow}\limits^{a}$ if no such states exist
- If $\sigma = a_1\ldots a_n$ for $a_1,\ldots ,a_n\in \textit{Act}$, we write $s\mathop {\Rightarrow }\limits ^{\sigma } s'$ if there are states $s_1, \ldots , s_{n-1}\in S$ such that $s\mathop {\Rightarrow }\limits ^{a_1}s_1\ldots s_{n-1}\mathop {\Rightarrow }\limits ^{a_n}s'$. We write $s\mathop {\Rightarrow }\limits ^{\sigma }$ if there is a *s'∈ S* so that $s\mathop {\Rightarrow }\limits ^{\sigma }s'$, and we write $s \mathop{\not\Rightarrow}\limits^{\sigma }$ if no such states exist
- We write $$\textit{traces}(s) = \{\sigma \in \textit{Act}^* \mid \ s\mathop {\Rightarrow }\limits ^{\sigma }\}$$ and $$\textit{traces}({\mathcal {A}})=$$$$\textit{traces}(s_0)$$
- We let *⊑ * denote the prefix relation on traces. If $\sigma ,\sigma '\in \textit{Act}^*$, $\sigma =a_1\ldots a_n$ and $\sigma '=a_1\ldots a_i$ for some *i≤ n* we write $\sigma '\sqsubseteq \sigma$ to denote *σ '* as subtrace of *σ *.

Throughout the paper, we use the suffix *-?* for inputs and *-!* for outputs, but these suffixes are not technically part of the label itself. Also, we use *i?* and *i*’*?* to denote inputs, and *o!* and *o*’*!* for outputs. We point out that *→ * includes *τ * labels, while *⇒ * does not (bullet 3 of the above definition).

Morever, in this paper, we exclude LTS with *τ * loops or cycles. For a treatise on the fundamental difference between *τ * loops (a.k.a. infinite unobservable internal progress) and absence of outputs (a.k.a. quiescence) the reader is referred to [6].

**Input–output transition system (IOTSs).** Besides using an LTS as a specification of expected behaviour for model-based testing, we assume that the actual behaviour of the SUT, the implementation, can be represented as an LTS as well. In **ioco** theory, the implementation is required to accepts all inputs at all times, i.e. each of its states is *input-enabled*. We call an input-enabled LTS an *input–output transition system* (IOTS).

### Definition 2

(*Input–Output Transition System*) An input–output transition system (IOTS) is an input-enabled LTS, i.e. for all $i? \in \textit{Act}_I$ and all *s∈ S*, we have $s\mathop {\Rightarrow }\limits ^{i?}$.

### Example 1

**Fig. 1:**
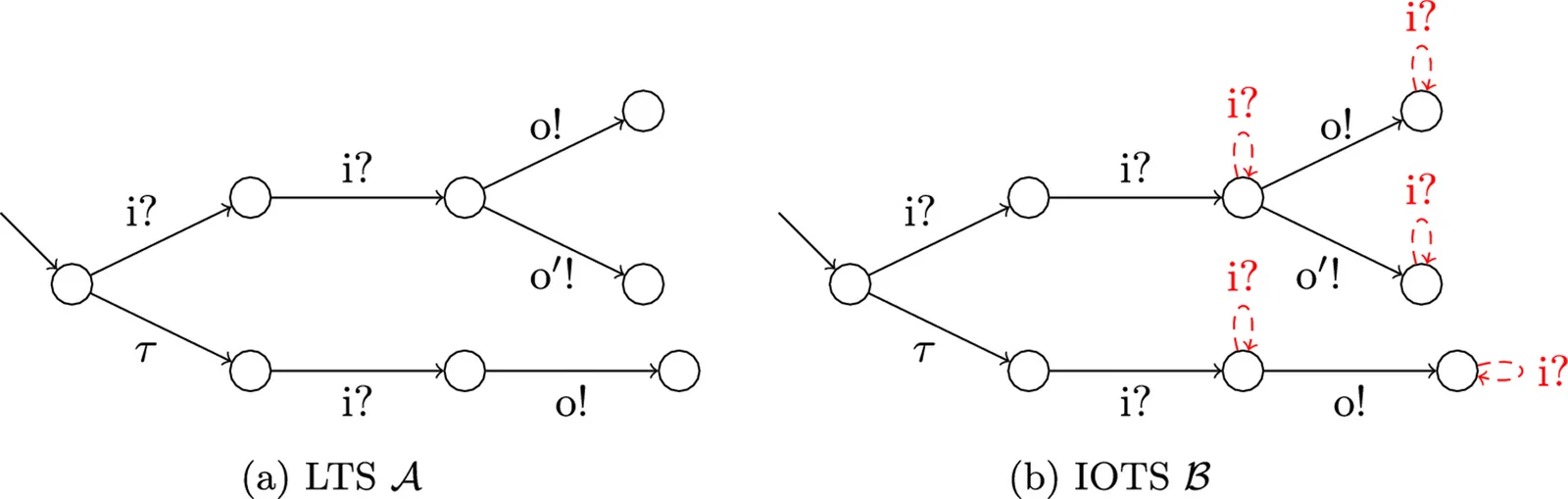
LTS ${\mathcal {A}}= \langle S,\{$i?,o!,o′!$\},\rightarrow _{\mathcal {A}},s_0\rangle$ and its input-enabled variant IOTS ${\mathcal {B}}= \langle S,\{$i?,o!,o′!$\},\rightarrow _{\mathcal {B}},s_0\rangle$

Figure 1 shows an example LTS ${\mathbf{\mathcal {A}}}$, and IOTS ${\mathbf{\mathcal {B}}},$ such that ${\mathbf{\mathcal {B}}}$ is an input-enabled version of ${\mathbf{\mathcal {A}}}$. The newly added input transitions are highlighted with dashed, red arrows.

**Quiescence.** Traces represent the *visible* behaviour of a labelled transition system. A state that does not have any outgoing output transitions is *quiescent*. Quiescent behaviour of a state is made explicit by adding a *δ * action that loops back to the same state.

### Definition 3

(*Quiescence*) A state *s∈ S* is *quiescent* iff

$$\forall \ o! \in \textit{Act}_O: s\mathop{\not\Rightarrow}\limits^{o!}$$

We write $\textit{Act}^\delta = \textit{Act}\cup \{\delta \}$, and $\textit{Act}_O^\delta = \textit{Act}_O\cup \{\delta \}$.

### Example 2

**Fig. 2:**
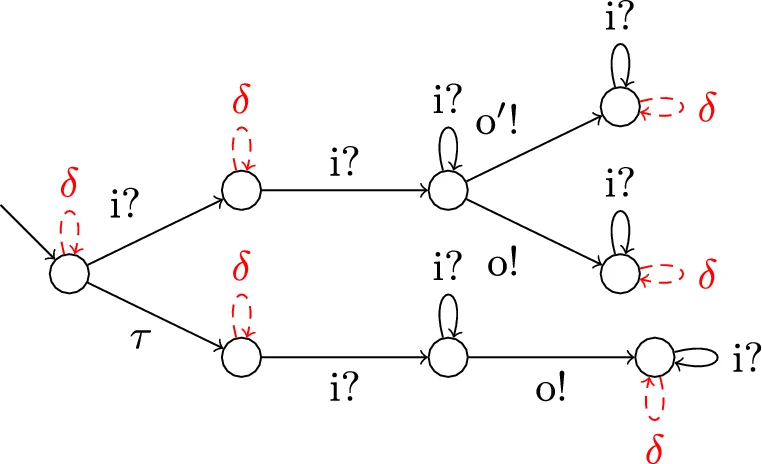
System ${\mathcal {B}}^\delta$ with its quiescent states highlighted by *δ *-self-loops

Figure 2 shows ${\mathbf{\mathcal {B}}}$, the IOTS from Fig. 1b, where the quiescent states are highlighted by *δ *-self-loops.

**Ioco.** The input–output conformance (**ioco**) relation formally defines when an implementation conforms to a specification. As stated, implementation and specification are modelled as labelled transition systems with inputs and outputs. Precisely, an implementation conforms to a specification if, after every observable trace allowed by the specification, the implementation can only produce outputs that are also permitted by the specification.

Suspension traces extend regular traces with the special quiescence action *δ * in quiescent states. These suspension traces are then used to define the **ioco** relation [1], where *δ * is considered among the outputs that an LTS/IOTS may produce. Definition 4 introduces the necessary notation for the definition of **ioco** in Definition 5.

### Definition 4

(**ioco** notation) Let ${\mathcal {A}}=\langle S, \textit{Act}_\tau , \rightarrow , s_0\rangle$ be an LTS. Below are **ioco** specific notations:

- All output actions, including *δ *, enabled in a state *s∈ S* are: $\mathbf{{out}}(s)= \left\{ o\in \textit{Act}_O{\ |\ }s\mathop {\Rightarrow }\limits ^{o}\right\} \cup \{\delta {\ |\ }\text {if } s\text { is quiescent}\}$
- All input actions enabled in a state *s∈ S* are: $\mathbf{{in}}(s) = \left\{ i\in \textit{Act}_I{\ |\ }s\mathop {\Rightarrow }\limits ^{i}\right\}$
- Let *ɛ * be the empty sequence, $a\in \textit{Act}^\delta$ an action or *δ *, and $\sigma \in (\textit{Act}^\delta )^*$ a sequence of actions, including *δ *. Then states after these sequences, starting in *s∈ S*, are:
  $$\begin{aligned}s\;{\mathbf {after}}\;\varepsilon &= \{ s\}\\s\;{\mathbf {after}}\;a & = \left\{ s' \mid a\in \textit{Act}\wedge s\mathop {\Rightarrow }\limits ^{a} s'\right\} \cup \left\{ s\mid a\right. \left. \quad = \delta \wedge s\text { is quiescent} \right\} \\s\;{\mathbf {after}}\;a\,\sigma &= \bigcup \ \left\{ s' \;{\mathbf {after}}\;\sigma \mid s'\in s\;{\mathbf {after}}\;a\right\} \end{aligned}$$
- The suspension traces of a state *s∈ S*, i.e. traces including quiescence, are: $$ Straces(s) = \left\{ {\sigma \in \left( {Act^{\delta } } \right)^{*}{\mid }s\;{\mathbf{after}}} \right. $$$$ \left. {\sigma \ne \emptyset } \right\} $$
- For an LTS ${\mathbf{\mathcal {A}}}$ we write: ${\mathcal {A}}\;{\mathbf {after}}\;\sigma =s_0\;{\mathbf {after}}\;\sigma$, and $\textit{Straces}({\mathcal {A}}) = \textit{Straces}(s_0)$.

We are now ready to recall classic input–output conformance (**ioco**) [1]. This relation defines when an IOTS implementation conforms to an LTS specification.

### Definition 5

(**ioco**) Let ${\mathbf{\mathcal {A}}}_I$ be an IOTS and ${\mathbf{\mathcal {A}}}_S$ an LTS. Then ${\mathbf{\mathcal {A}}}_I\ {\mathbf {ioco}}\ {\mathbf{\mathcal {A}}}_S$ iff

$$ \forall \ \sigma \in \textit{Straces}({\mathbf{\mathcal {A}}}_S): \mathbf{{out}}\left( {\mathbf{\mathcal {A}}}_I\;{\mathbf {after}}\;\sigma \right) \subseteq \mathbf{{out}}\left( {\mathbf{\mathcal {A}}}_S\;{\mathbf {after}}\;\sigma \right) . $$

### Example 3

**Fig. 3:**
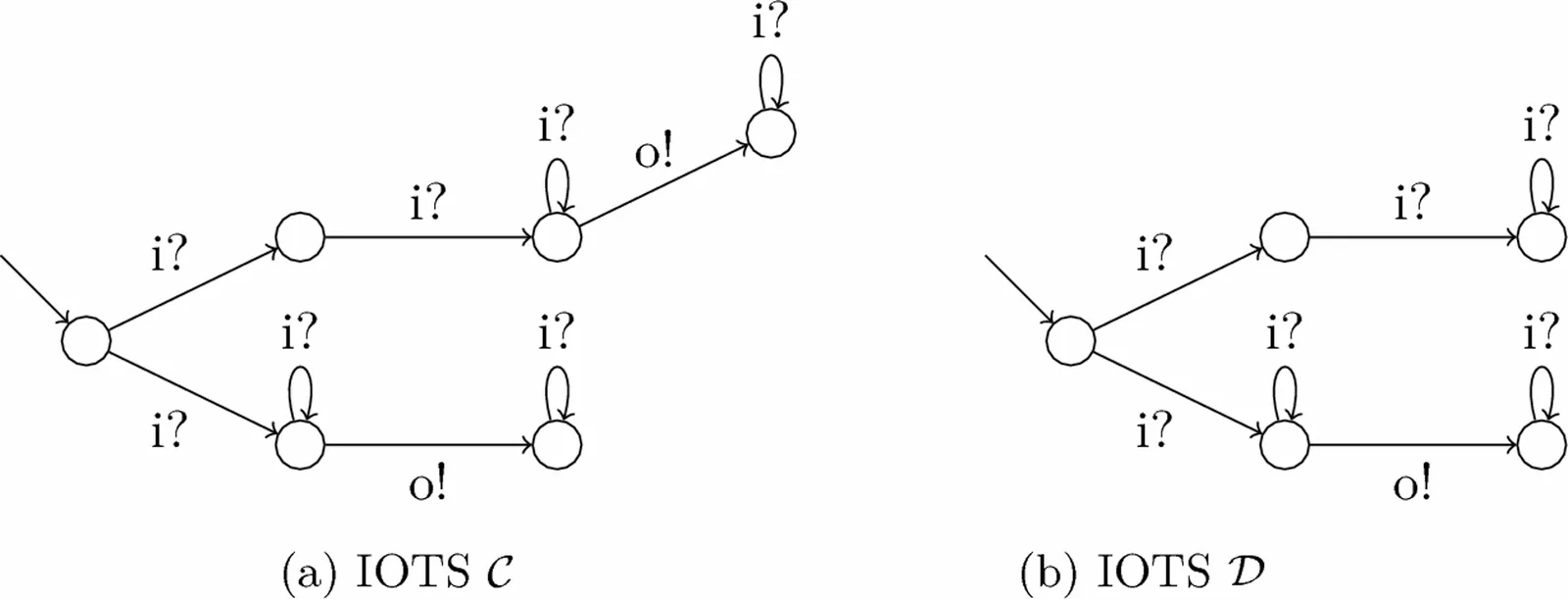
IOTS $${\mathcal {C}}= \langle S,\{$$i?,o!,o′!$$\},\rightarrow ,s_0\rangle $$ and IOTS $${\mathcal {D}}= \langle S,\{$$i?,o!,o′!$$\},\rightarrow ,s_0\rangle $$. We have $${\mathcal {C}}\ {\mathbf {ioco}}\ {\mathcal {A}}$$, but $${\mathcal {D}}$$$$\ /\!\!\!\!\!\!{\mathbf {ioco}}\ {\mathcal {A}}$$

In Fig. 3, we have one implementation $${\mathcal {C}}\in $$ IOTS and on the right we have another implementation $${\mathcal {D}}\in $$ IOTS of the specification $${\mathcal {A}}\in $$ LTS from Fig. 1a. We observe that $${\mathcal {C}}\ {\mathbf {ioco}}\ {\mathcal {A}}$$, but $${\mathcal {D}}$$$$\ /\!\!\!\!\!\!{\mathbf {ioco}}\ {\mathcal {A}}$$, because of the suspension trace $$\sigma = i? \cdot \delta \cdot i?$$, where $$\delta \in \mathbf{{out}}({\mathcal {D}}\;{\mathbf {after}}\;\sigma )$$ but $$\delta \not \in \mathbf{{out}}({\mathcal {A}}\;{\mathbf {after}}\;\sigma )$$.

## Timed Automata and $\mathbf{tioco}_M$

Inspired by [7] we define a timed automaton (TA) as a labelled transition system with clock variables, clock constraints on states (now called locations), and transitions with clock guards. Again, we split actions into inputs and outputs. We first define notation for clock constraints. Throughout, the time domain for clocks we consider is ${\mathbb {R}}_{\ge 0}$, i.e. the set of nonnegative real numbers.

### Definition 6

(*Clock constraint*) The set of clock constraints over a set of clock variables ${\mathcal {C}}$ is $\Phi ({\mathcal {C}})$, defined as in [7]. In particular a *clock constraint*$\pi \in \Phi ({\mathcal {C}})$ is an element from grammar:

$$\begin{aligned} \phi := c\le {\mathbf{K}}\mid {\mathbf{K}}\le c\mid c< {\mathbf{K}}\mid {\mathbf{K}}< c\mid \phi _1 \wedge \phi _2 \end{aligned}$$

for any clock variables $c\in {\mathcal {C}}$ and constants ${\mathbf{K}}\in {\mathbb {R}}_{\ge 0}$.

As is standard for a TA, a state consists of a location paired with a clock valuation, with all clocks advancing at the same rate. In our setting, we use only a single clock, so this general multi-clock mechanism is not required.

### Definition 7

(*Timed Automaton*) A *timed automaton* (TA) ${\mathscr {A}}$ is a tuple $\langle L, \textit{Act}_\tau , \Phi _L, {\mathcal {C}}, \rightarrow ,\ell _0\rangle$, where

- *L* is a finite set of locations with $\ell _0\in L$ as the initial location
- $$\textit{Act}_\tau $$ is the finite set of action labels subdivided in input and output actions and the *τ * action, i.e. $$\textit{Act}_\tau = \textit{Act}\cup \{\tau \},$$$$\textit{Act}= \textit{Act}_I\cup \textit{Act}_O$$ and $$\textit{Act}_I\cap \textit{Act}_O= \emptyset $$
- ${\mathcal {C}}$ is a finite set of clock variables
- $\Phi _L: L\rightarrow \Phi ({\mathcal {C}})$ is a function that maps each location $\ell \in L$ to some clock constraint in $\Phi ({\mathcal {C}})$. We call $\Phi _L(\ell )$ the *invariant* of *ℓ *
- $\rightarrow \ \subseteq L\times \textit{Act}_\tau \times \Phi ({\mathcal {C}})\times 2^{{\mathcal {C}}}\times L$ is a set of timed transitions. A timed transition $\langle \ell , a, \phi , \lambda , \ell '\rangle \in \ \rightarrow$ represents an edge from location *ℓ * to location *ℓ '*, on action *a* (including *τ *). With $\phi \in \Phi ({\mathcal {C}})$ we denote its clock constraint, called *guard*, which specifies when the transition is enabled. The set $\lambda \subseteq {\mathcal {C}}$ gives the clocks to be reset, i.e. set to value 0, with this transition.

As per usual, *locations* in a TA differ from *states*. Commonly, the latter is only used when we talk about the TA’s semantics, i.e. a location is a tuple of a state and a clock evaluation. Consequently, a TA’s semantics has uncountably many states based on the uncountably many non-negative clock valuations. As is common, we exclude *Zeno* behaviour, i.e. infinitely many actions happening in a finite amount of time.

Traces in timed automata are sequences of *(d,a)*-tuples, denoting that *a* happens after the passage of *d* time units in the previous location. The invariants and guards of the TA specify whether the transition can be taken after *d* time.

### Definition 8

(*TA Notation*) Let ${\mathscr {A}}=\langle L, \textit{Act}_\tau , \Phi _L, {\mathcal {C}}, \rightarrow ,\ell _0\rangle$ be a TA:

- We write $\ell \xrightarrow {(d,a)}\ell '$ for $(d,a) \in {\mathbb {R}}_{\ge 0}\times \textit{Act}_\tau$ if there is a $\langle \ell , a, \phi , \lambda , \ell '\rangle \in \ \rightarrow$ such that *ϕ * holds at time *d*, and $\Phi _L(\ell )$ holds from time 0 (the time of arrival in *ℓ *) to time *d*, and such that $\Phi _L(\ell ')$ is true after updating the clocks with duration *d* and the resets from *λ *.
- We write $\ell \mathop {\Rightarrow }\limits ^{(d,\epsilon )}\ell '$ if either $\ell = \ell '$ and *d=0*, or $\ell \xrightarrow {(d_1,\tau )\ldots (d_n,\tau )}\ell '$ and $d=d_1+\dots +d_n$. We write $\ell \mathop {\Rightarrow }\limits ^{(d,a)}\ell '$ for any $a\in \textit{Act}$ if there are $s_1,s_2\in S$ such that $\ell \mathop {\Rightarrow }\limits ^{(d_1,\epsilon )}s_1\xrightarrow {(d_2,a)}s_2\mathop {\Rightarrow }\limits ^{(d_3,\epsilon )}\ell '$ for $d = d_1+d_2+d_3$.
- Timed traces are sequences of non-negative numbers and visible actions, i.e. $$\textit{ttraces}(\ell ) =$$$$ \{\rho \in ({\mathbb {R}}_{\ge 0}\times \textit{Act})^{*} {\ |\ }\ell \mathop {\Rightarrow }\limits ^{\rho }\}$$
- Other notations on traces and subtraces carry over straightforwardly from Definition 1.

To define $\mathbf{tioco}_M$ [4], the timed variant of **ioco**, we introduce some additional notation. We explicitly use a duration parameter $M\in {\mathbb {R}}_{>0}$ after which we declare quiescence.

### Definition 9

(*tioco*_*M*_* notation*) Let ${\mathscr {A}}=\langle L, \textit{Act}_\tau , \Phi _L, {\mathcal {C}}, \rightarrow ,\ell _0\rangle$ be a TA, and let $M\in {\mathbb {R}}_{>0}$ be the time to declare quiescence explicitly:

- A location $\ell \in L$ is *quiescent* iff
  $$\forall \ {\textit{d}}\in {\mathbb {R}}_{\ge 0}, \forall \ o! \in \textit{Act}_O: {\mathrm{if}} d < M \text { then } \ell \ \mathop{\not\Rightarrow}\limits^{({\textit{d}},o!)}$$
- The outputs or inputs enabled in a location *ℓ * are given as:
  $$\begin{aligned}\mathbf{{out}}_M(\ell ) &= \left\{ ({\textit{d}},o!)\in {\mathbb {R}}_{\ge 0}\times \textit{Act}_O{\ |\ }\ell \mathop {\Rightarrow }\limits ^{({\textit{d}},o!)}\right\} \cup \left\{ (M,\delta ){\ |\ }\ell \text { is quiescent}\right\} \\\mathbf{{out}}(\ell ) &= \left\{ o! \in \textit{Act}_O^\delta {\ |\ }\exists \ ({\textit{d}}, o!) \in \mathbf{{out}}_M(\ell ) \right\} \\\mathbf{{in}}(\ell ) &= \left\{ i? \in \textit{Act}_I{\ |\ }\exists \ ({\textit{d}}, i?) \in {\mathbb {R}}_{\ge 0}\times \textit{Act}_I\wedge \ell \mathop {\Rightarrow }\limits ^{({\textit{d}},i?)}\right\} \end{aligned}$$
- We define locations after a sequence of tuples of a time duration *d* and action *a*, including the tuple *(M,δ )* for quiescent locations, starting in location : $$\ell \;\mathbf{after}_M\;\epsilon $$ = $$\{ \ell \}$$$$\ell \;\mathbf{after}_M\;({\textit{d}},a)$$ = $$\{\ell ' \mid a\in \textit{Act}\wedge \ \ell \mathop {\Rightarrow }\limits ^{({\textit{d}},a)} \ell '\}\ \cup $$$$\{\ell \mid ({\textit{d}},a) = (M,\delta ) \wedge \ \ell \text { is quiescent} \}$$$$\ell \;\mathbf{after}_M\;({\textit{d}},a)\rho $$ = $$\bigcup \ \{\ell ' \;\mathbf{after}_M\;\rho \mid \ell '\in \ell \;\mathbf{after}_M\;({\textit{d}},a)\}$$  
- We define the suspension timed traces as the traces of location *ℓ *, including *δ * at time *M*, for quiescent locations encountered in the trace: $\textit{Sttraces}_M(\ell ) = \{\rho \in ({\mathbb {R}}_{\ge 0}\times \textit{Act}^\delta )^* \mid \ell \;\mathbf{after}_M\;\rho \ne \emptyset \}$
- We write: $${\mathscr {A}}\;{\mathbf {after}}\;\rho =\ell _0\;{\mathbf {after}}\;\rho $$ and $$\textit{Sttraces}_M({\mathscr {A}})=$$$$\textit{Sttraces}_M(\ell _0)$$.

Lastly, for testing purposes, we define input enabled TAs called input–output timed automata (IOTA).

### Definition 10

(*Input–Output Timed Automaton*) An input–output timed automaton (IOTA) is an input-enabled TA for parameter $M\in {\mathbb {R}}_{>0},$ i.e.

$$\begin{aligned} \forall \ d < M \in {\mathbb {R}}_{\ge 0}, \forall \ i? \in \textit{Act}_I, \forall \ \ell \in L: \ell \mathop {\Rightarrow }\limits ^{(d,i?)}. \end{aligned}$$

We will now define timed input–output conformance $\mathbf{tioco}_M,$ as in [8]. Note that $\textit{Sttraces}_M({\mathscr {A}}_S)$ only contains the quiescence label *δ * at *exactlyM* time units.

### Definition 11

($\mathbf{tioco}_M$) Let ${\mathscr {A}}_I$ be an IOTA, ${\mathscr {A}}_S$ be a TA and $M \in {\mathbb {R}}_{>0}$, then we define ${\mathscr {A}}_I\ \mathbf{tioco}_M\ {\mathscr {A}}_S$ iff

$$\begin{aligned} & \forall \ \rho \in \textit{Sttraces}_M\left( {\mathscr {A}}_S\right) : \mathbf{{out}}_M\left( {\mathscr {A}}_I\;\mathbf{after}_M\;\rho \right) \subseteq \mathbf{{out}}_M\left( {\mathscr {A}}_S\;\mathbf{after}_M\;\rho \right) . \end{aligned}$$

## Transformations

We show that **ioco** is preserved when going from LTS/IOTS to TA/IOTA. Below we define the transformation from an LTS to a TA. Definition 12 is central to our contribution: it details the conversion of an LTS into a TA in which quiescence is represented by a dedicated transition labelled *δ * that becomes enabled after a time-out *M*, whereas other output transitions are restricted to the *t < M*.

Introducing the notion of time naturally implies the addition of clocks. Since LTSs do not inherently have a notion of time, the only clock *c* that is necessary to add is the one to measure quiescence. The parameter $M\in {\mathbb {R}}_{>0}$ is the explicit time when we declare a location as quiescent. Naturally, each output transition gets a guard *c<M* to ensure it is taken strictly before *M* time units have passed. The only enabled output at exactly *M* time units is the quiescent transition *δ *. To ensure that either an output or *δ * is observed, either before *M* time units have passed (when an output is observed), or when exactly *M* time units have been observed (when quiescence is observed), we add the invariant *c ≤ M* to each location. Inputs also need to be provided strictly before *M*, because it is not needed to wait for quiescence before providing the input. Inputs, outputs and quiescence represent the *visible* behaviour of the system, and quiescence measures the time passed since the last visible progress. Thus, like a stopwatch, the clock *c* is reset on every visible action. This closely reflects how testing of timed systems is done in practice. Our work provides the mathematical underpinning of its correctness.

### Definition 12

(*TA-ification*) The TA-ification of an LTS,

${\mathcal {A}}=\langle S, \textit{Act}_\tau , \rightarrow , s_0\rangle$, for parameter $M\in {\mathbb {R}}_{>0}$ is a function $\chi ^{\scriptstyle M}\!$: $\textit{LTS}\rightarrow \textit{TA}$, with ${\mathscr {A}}= \langle L, \textit{Act}_\tau ^\delta , \Phi _L, \{c\}, \rightarrow _{\mathscr {A}}, s_0 \rangle$ such that:

1. States *S*, including initial state $s_0,$ identify locations of ${\mathscr {A}},$ i.e. *L= S*.
2. $\textit{Act}_\tau ^\delta$ are the actions labels of ${\mathscr {A}}$ (i.e. all actions of ${\mathbf{\mathcal {A}}}$ and additionally *δ *)
3. *c* is the unique clock of ${\mathscr {A}}$ used to track quiescence
4. $\Phi _L:L\rightarrow \Phi ({\mathcal {C}})$ is a function assigning clock constraints to ${\mathscr {A}}$’s locations as follows: for all *l ∈ L* we have $\Phi _L(\ell ) = c\le M$
5. $\rightarrow _{{\mathscr {A}}}: L\times \textit{Act}_\tau \times L$ defines ${\mathscr {A}}'s$ transition relation as an extension of *→ * with clock constraints and resets, as follows:
  $$\begin{aligned} \rightarrow _{{\mathscr {A}}}\ =&\{(\ell ,a,\{c< M\},\{c\},\ell ') \mid (\ell ,a,\ell ') \in \ \rightarrow ,\ a\in \textit{Act}\}\ \cup \\&\{(\ell , \delta ,\{c= M\},\{c\},\ell ) \mid \ell \in L\text { is quiescent}\}\ \cup \\&\{(\ell , \tau ,\{\},\{\},\ell ') \mid (\ell ,\tau ,\ell ') \in \ \rightarrow \} \end{aligned}$$
  For an LTS ${\mathbf{\mathcal {A}}}$ we call its resulting TA, i.e. $\chi ^{\scriptstyle M}\!({\mathcal {A}}) = {\mathscr {A}}$, the *canonic* TA of ${\mathbf{\mathcal {A}}}$.

We depict the TA-ification in Table 1. It shows the TA-ification of a single transition, disregarding the other transitions of its source location.

**Table 1:**
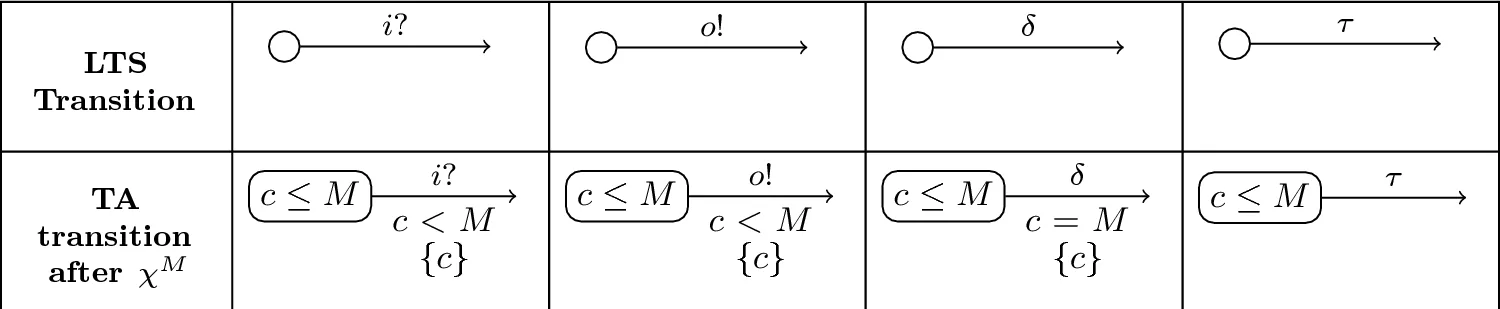
Visual representation of $\chi ^{\scriptstyle M}\!$ (cf. Definition 12). All locations get an invariant and their transitions get an appropriate guard to enforce that *δ * can *only* be observed after *M* time units

### Example 4

**Fig. 4:**
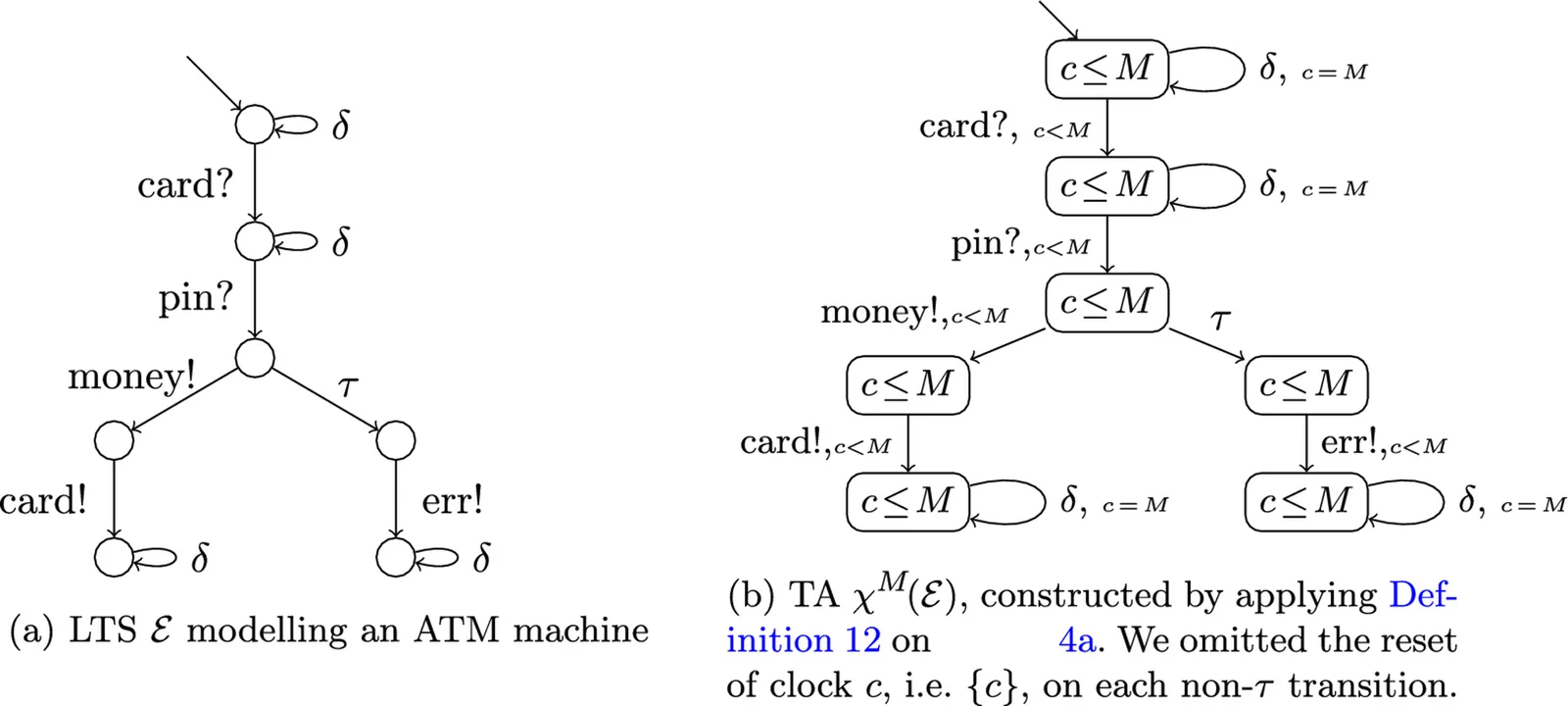
Transformation of an LTS ${\mathcal {E}}= \langle S,\{$card?,pin?,money!,card!,err!$\},\rightarrow ,s_0\rangle$ into a TA with a TA-ification $\chi ^{\scriptstyle M}\!({\mathcal {E}})$

Consider the LTS ${\mathcal {E}}$ and its corresponding TA $\chi ^{\scriptstyle M}\!({\mathcal {E}})$ presented in Fig. 4. The system receives a card and a PIN. Then, it dispenses money and returns the card. Otherwise, it displays an error message.

After the transformation from states to locations, each state with an output-outgoing transitions was decorated with a *c≤ M* invariant and all visible output-outgoing transitions are annotated with a *c<M* guard and a clock reset *c*. In this manner we ensure that any visible output strictly occurs before *M* time units.

For proving conformance preservation of our $\chi ^{\scriptstyle M}\!$ operator, we translate back from $\chi ^{\scriptstyle M}\!({\mathcal {A}})$ to ${\mathbf{\mathcal {A}}}$. When we formally want to disregard the timing aspects (i.e. guards, invariants, clocks) it is useful to formally define a TA’s *projection* onto its untimed system.

### Definition 13

(*Projection*) Let ${\mathscr {A}}_S=\langle L, \textit{Act}_\tau , \Phi _L, {\mathcal {C}}, \rightarrow ,\ell _0\rangle$ be a TA. Then its *projected LTS* is: ${\mathbf{\mathcal {A}}}_S= \langle L, \textit{Act}_\tau , \rightarrow ', \ell _0 \rangle,$ where:

$$\begin{aligned} \rightarrow '\; = \left\{ \langle \ell ,a,\ell '\rangle \in S\times \textit{Act}_\tau \times S \mid \langle \ell , a, \phi , \lambda , \ell '\rangle \in \rightarrow \right\} \end{aligned}$$

Let $\rho =(d_1,a_1)\ldots (d_n,a_n) \in \textit{Sttraces}({\mathscr {A}}_S)$ be a suspended timed trace. We define the *projection* of $\sigma = a_1 \ldots a_n,$ i.e. its regular suspended trace without time, as $[\rho ]\downarrow \; = \sigma$.

The following lemma describes the relation between traces of an LTS and timed traces in its canonic TA. In essence, we may disregard the time-label associated to each action since the transformation $\chi ^{\scriptstyle M}\!$ neither adds nor removes behaviour, e.g. we associate $(d_1,a_1)\ldots (d_n,a_n)$ with $a_1\ldots a_n$ for some $d_i\in {\mathbb {R}}_{>0}$.

### Lemma 1

(Traces of Canonic TAs) Let ${\mathcal {A}}=\langle S, \textit{Act}_\tau , \rightarrow , s_0\rangle$ be an LTS and $M\in {\mathbb {R}}_{>0}$, then:

1. If $\sigma \in \textit{Straces}({\mathcal {A}})$, then there is $\rho \in \textit{Sttraces}_M(\chi ^{\scriptstyle M}\!({\mathcal {A}}))$ such that $[\rho ]\downarrow =\sigma$
2. If $\rho \in \textit{Sttraces}_M(\chi ^{\scriptstyle M}\!({\mathcal {A}}))$, then there is $\sigma \in \textit{Straces}({\mathcal {A}})$ such that $[\rho ]\downarrow =\sigma$.

### Proof

$\boxed {Case\,1}$ If $\sigma \in \textit{Straces}({\mathcal {A}})$ we need to show that for given $M\in {\mathbb {R}}_{>0}$ there exists $\rho \in \textit{Sttraces}_M(\chi ^{\scriptstyle M}\!({\mathcal {A}}))$ such that $[\rho ]\downarrow =\sigma$ (cf. Definition 13). The proof is by induction on the trace length of *σ *, i.e. on *|σ |*.

*Base case.* Consider the empty trace $\sigma =\varepsilon$ with trace length zero, i.e. *|σ |=0*. With Definition 12$\chi ^{\scriptstyle M}\!({\mathcal {A}})$ is a timed automaton with initial location $\ell _0$, and with no visible action transition taken, the projection of any empty (suspended) timed trace $\rho \in \textit{Sttraces}_M(\chi ^{\scriptstyle M}\!({\mathcal {A}}))$ is also empty, i.e. $[\rho ]\downarrow =\varepsilon =\sigma$. Thus, the claim holds for *|σ |*.

*Induction hypothesis.* Assume that the claim holds for traces of length *n* for some $n\in {\mathbb {N}}$. We now show that the claim also holds for traces of length *n+1*.

*Induction Step.* Assume $\sigma '\in \textit{Straces}({\mathcal {A}})$ with *|σ '|=n+1.* Thus, there are $\sigma \in \textit{Straces}({\mathcal {A}})$ with *|σ |=n* and $a\in \textit{Act}^\delta$ such that we may also write $\sigma '=\sigma \cdot a$. By definition of traces (cf. Definition 1)—and by trivial extension to suspended traces—there are states $s_i\in S$ such that:

$$\begin{aligned} s_0\mathop {\Rightarrow }\limits ^{a_1}s_1\mathop {\Rightarrow }\limits ^{a_2}\ldots \mathop {\Rightarrow }\limits ^{a_n}s_n\mathop {\Rightarrow }\limits ^{a}s_{n+1}, \text {or equivalently} \end{aligned}$$

$$\begin{aligned} s_0\mathop {\Rightarrow }\limits ^{\sigma }s_n\mathop {\Rightarrow }\limits ^{a}s_{n+1}, \text { with } \sigma = a_1 \cdots a_n. \end{aligned}$$

According to the induction hypothesis, there is a (suspended) timed trace $\rho \in \textit{Sttraces}_M(\chi ^{\scriptstyle M}\!({\mathcal {A}}))$ with $[\rho ]\downarrow =\sigma$. Moreover we know $\ell _n \;\mathbf{after}_M\;\rho$ as the equivalent location in $\chi ^{\scriptstyle M}\!({\mathcal {A}})$ to the state $s_n$ (since each $s_i$ identifies $\ell _i$ directly). From here it suffices to show that for the last visible action of the trace in the LTS, there is the same action in the timed automaton for some guard $g\in \Phi$ and clock reset $c\subseteq {\mathcal {C}}$. We distinguish the three cases: $a=i?\in \textit{Act}_I$, $a=o!\in \textit{Act}_O$ and *δ *:

$\boxed {a=i?}$ According to the transformation (cf. Definition 12) this means that there is time $d\in {\mathbb {R}}_{\ge 0}$ such that $\ell _n\mathop {\Rightarrow }\limits ^{(d,i?)}\ell _{n+1}$. Also, in $s_n$ there is either

1. $s_n \mathop {\Rightarrow }\limits ^{o!}$, or there is
2. $s_n \mathop{\not\Rightarrow}\limits^{o!}$, in which case the state is quiescent and a *δ * is added in the suspension traces by Definition 3.

In either case, due to the transformation rules (cf. Definition 12) there is a clock invariant *c≤ M* in $\ell _n$. This means that, for all *0≤ d<M* there is a transition:

$$\begin{aligned} \ell _n\mathop {\Rightarrow }\limits ^{(d,i?)}\ell _{n+1} \end{aligned}$$

$\boxed {a=o!}$ With Definition 12 there is a $d\in {\mathbb {R}}_{\ge 0}$ such that $\ell _n\mathop {\Rightarrow }\limits ^{(d,o!)}\ell _{n+1}.$ In particular, there is a clock invariant *c≤ M* in $\ell _n$. This means that, for all *0≤ d<M* there is a transition

$$\begin{aligned} \ell _n\mathop {\Rightarrow }\limits ^{(d,o!)}\ell _{n+1} \end{aligned}$$

$\boxed {\delta }$ With Definition 12 there is $\ell _n\mathop {\Rightarrow }\limits ^{(M,\delta )}\ell _{n+1}$. In particular, there is a clock invariant *c≤ M* in $\ell _n$. This means there is a transition

$$\begin{aligned} \ell _n\mathop {\Rightarrow }\limits ^{(M,\delta )}\ell _{n+1} \end{aligned}$$

In each case (depending on input, output or *δ )* we can choose $0\le d\le M$ such that $\rho '=\rho \cdot ({\textit{d}},a)\in \textit{Sttraces}_M(\chi ^{\scriptstyle M}\!({\mathcal {A}}))$. Moreover $[\rho ']\downarrow =\sigma '$ by construction, which concludes the induction.

$$\boxed {Case\, 2}$$ The proof is by construction. Let $$\rho \in $$$$\textit{Sttraces}_M(\chi ^{\scriptstyle M}\!({\mathcal {A}}))$$. Then we need to find $$\sigma \in \textit{Straces}({\mathcal {A}})$$ such that $$[\rho ]\downarrow =\sigma $$. With Definition 8 any suspension timed traces can be written as:

$$\begin{aligned} \ell _1 \mathop {\Rightarrow }\limits ^{(d_1,a_1)}\ell _2 \mathop {\Rightarrow }\limits ^{(d_2,a_2)}\ldots \mathop {\Rightarrow }\limits ^{(d_{n-1},a_{n-1})}\ell _n \end{aligned}$$

In particular, with the definition of the transformation (cf. Definition 12) each transition in $\rightarrow _{\chi ^{\scriptstyle M}\!({\mathcal {A}})}$ originates from an LTS transition, or is an explicitly added *δ * self-loop in quiescent states. The TA-ification only extends the original discrete transitions with additional guards and resets. More precisely, by Definition 12 every transition in $\rightarrow _{\chi ^{\scriptstyle M}\!({\mathcal {A}})}$ is one of the three forms for *a= i?*, *a= o!* and *a= δ *:

- $(\ell , i?, c<M, \{c\},\ell ')$ for inputs $(s, i?, s')\in \ \rightarrow _{{\mathcal {A}}}$ and $i?\in \textit{Act}_I$
- $(\ell , o!, c< M, \{c\},\ell ')$ for outputs $(s, o!, s')\in \ \rightarrow _{{\mathcal {A}}}$ and $o!\in \textit{Act}_O$
- $(\ell , \delta , c=M, \{c\},\ell )$ for quiescent states, i.e. *(s,δ ,s)* in suspended traces
- $(\ell , \tau , \{\}, \{\},\ell ')$ for internal actions i.e. $(s,\tau ,s')\in \ \rightarrow _{{\mathcal {A}}}$

In each case, according to Definition 13, the projection $[\rho ]\downarrow$ removes the information of the transition that is only relevant for TAs and not in LTSs (i.e. delays $d\in {\mathbb {R}}_{\ge 0}$), and we are left with the suspended trace

$$\begin{aligned} \sigma = a_1 a_2 \ldots a_n\in \textit{Straces}({\mathcal {A}}) \end{aligned}$$

Consequently, $[\rho ]\downarrow =\sigma$, which concludes the proof.

*□*

With Lemma 1 every original transition of an LTS is preserved under the transformation to a TA. Hence, it easily follows that the transformation also preserves input–enabledness.

### Corollary 2

Let ${\mathbf{\mathcal {A}}}$ be an IOTS and $M\in {\mathbb {R}}_{>0}$, then $\chi ^{\scriptstyle M}\!({\mathcal {A}})$ is an IOTA.

We are now able to state one of our paper’s main contributions: transformation of an LTS to a TA preserves conformance from **ioco** to $\mathbf{tioco}_M$.

### Theorem 3

Let ${\mathbf{\mathcal {A}}}_I$ be an IOTS and ${\mathbf{\mathcal {A}}}_S$ be an LTS. Then:

$$ {\mathbf{\mathcal {A}}}_I\ \mathbf{ioco}\ {\mathbf{\mathcal {A}}}_S\Longleftrightarrow \chi ^{\scriptstyle M}\!({\mathbf{\mathcal {A}}}_I)\ \mathbf{tioco}_M\ \chi ^{\scriptstyle M}\!({\mathbf{\mathcal {A}}}_S) $$

for all $$M\in {\mathbb {R}}_{>0}$$.

### Proof

$\boxed {\implies }$ Let ${\mathbf{\mathcal {A}}}_I$ be an IOTS, ${\mathbf{\mathcal {A}}}_S$ be an LTS and assume that ${\mathbf{\mathcal {A}}}_I\ \mathbf{ioco}\ {\mathbf{\mathcal {A}}}_S$.

Let $M\in {\mathbb {R}}_{>0}$, then we need to show that $\chi ^{\scriptstyle M}\!({\mathbf{\mathcal {A}}}_I)\ \mathbf{tioco}_M\ \chi ^{\scriptstyle M}\!({\mathbf{\mathcal {A}}}_S)$.

According to Definition 11, we need to show that for all suspension timed traces of the specification $\rho \in \textit{Sttraces}_M(\chi ^{\scriptstyle M}\!({\mathbf{\mathcal {A}}}_S))$ it holds that

$$ \mathbf{{out}}_M\left( \chi ^{\scriptstyle M}\!({\mathbf{\mathcal {A}}}_I)\;\mathbf{after}_M\;\rho \right) \subseteq \mathbf{{out}}_M\left( \chi ^{\scriptstyle M}\!({\mathbf{\mathcal {A}}}_S)\;\mathbf{after}_M\;\rho \right) $$

Thus, let $$\rho \in \textit{Sttraces}_M(\chi ^{\scriptstyle M}\!({\mathbf{\mathcal {A}}}_S))$$.

If $\rho \notin \textit{Sttraces}_M(\chi ^{\scriptstyle M}\!({\mathbf{\mathcal {A}}}_I))$ the inclusion is trivial because then $\mathbf{{out}}_M(\chi ^{\scriptstyle M}\!({\mathbf{\mathcal {A}}}_I)\;\mathbf{after}_M\;\rho )=\varnothing$. Otherwise, pick $({\textit{d}},o)\in \mathbf{{out}}_M(\chi ^{\scriptstyle M}\!({\mathbf{\mathcal {A}}}_I)\;\mathbf{after}_M\;\rho )$ with $d\in {\mathbb {R}}_{>0}$ and $o\in \textit{Act}_O^\delta$.

Then $$ \rho \!\cdot \!(d,o)=$$ and consequently $$\rho '\in$$$$\textit{Sttraces}(\chi ^{\scriptstyle M}\!({\mathbf{\mathcal {A}}}_I))$$.

It remains to be shown that $\rho '\in \textit{Sttraces}_M(\chi ^{\scriptstyle M}\!({\mathbf{\mathcal {A}}}_S))$ as this guarantees that $(d,o)\in \mathbf{{out}}_M(\chi ^{\scriptstyle M}\!({\mathbf{\mathcal {A}}}_S)\;\mathbf{after}_M\;\rho ).$

With Lemma 1 and the definition of the transformation (cf. Definition 12), there is an untimed trace $\sigma \in \textit{Straces}({\mathbf{\mathcal {A}}}_I)$ such that $[\rho ]\downarrow =\sigma$. By the same argument we can derive $\sigma '\in \textit{Straces}({\mathbf{\mathcal {A}}}_I)$ from *ρ '*, i.e.

with $o\in \textit{Act}_O^\delta$ and $\sigma '=\sigma \!\cdot \!o$ we again use the transformation (cf. Definition 12) and Lemma 1 to conclude that $\sigma '=\sigma \cdot o\in \textit{Straces}({\mathbf{\mathcal {A}}}_I)$.

Based on the premise ${\mathbf{\mathcal {A}}}_I\ \mathbf{ioco}\ {\mathbf{\mathcal {A}}}_S$ we know that

$$ \forall \ \xi \in \textit{Straces}\left( {\mathbf{\mathcal {A}}}_S\right) : \mathbf{{out}}\left( {\mathbf{\mathcal {A}}}_I\;{\mathbf {after}}\;\xi \right) \subseteq \mathbf{{out}}\left( {\mathbf{\mathcal {A}}}_S\;{\mathbf {after}}\;\xi \right) $$

and thus with $o\in \mathbf{{out}}({\mathbf{\mathcal {A}}}_I\;{\mathbf {after}}\;\sigma )\subseteq \mathbf{{out}}({\mathbf{\mathcal {A}}}_S\;{\mathbf {after}}\;\sigma )$ also $\sigma '\in \textit{Straces}({\mathbf{\mathcal {A}}}_S)$.

We are left to determine $d\in {\mathbb {R}}_{\ge 0}$.

For that we distinguish two cases: *o=δ * or $o\in \textit{Act}_O$.

- Assume *o=δ *. By Definition 12 the only guard possible is *ϕ =(c=M)*. Thus, the only available trace in $\textit{Sttraces}_M(\chi ^{\scriptstyle M}\!({\mathbf{\mathcal {A}}}_I))$ requires *d=M*. With Definition 9 this implies $(M,\delta )\in \mathbf{{out}}_M(\chi ^{\scriptstyle M}\!({\mathbf{\mathcal {A}}}_I) \;\mathbf{after}_M\;\rho )$. We conclude $(M,\delta )\in \mathbf{{out}}_M(\chi ^{\scriptstyle M}\!({\mathbf{\mathcal {A}}}_S) \;\mathbf{after}_M\;\rho )$.
- The case of $o\in \textit{Act}_O$ proceeds analogously, albeit with *ϕ =(c<M)* and some ${\textit{d}}<M$, again via the transformation rules of Definition 12.

Summarizing both cases, we conclude that there is a duration *d≤ M* such that $(d,o)\in \mathbf{{out}}_M(\chi ^{\scriptstyle M}\!({\mathbf{\mathcal {A}}}_S) \;\mathbf{after}_M\;\rho ).$ Since $M\in {\mathbb {R}}_{>0}$ was arbitrary but fixed, this shows that for all $\rho \in \textit{Sttraces}_M(\chi ^{\scriptstyle M}\!({\mathbf{\mathcal {A}}}_S))$:

$$ \mathbf{{out}}_M\left( \chi ^{\scriptstyle M}\!({\mathbf{\mathcal {A}}}_I) \;\mathbf{after}_M\;\rho \right) \subseteq \mathbf{{out}}_M\left( \chi ^{\scriptstyle M}\!({\mathbf{\mathcal {A}}}_S) \;\mathbf{after}_M\;\rho \right) . $$

In addition to this, we know via Theorem 2 that $\chi ^{\scriptstyle M}\!({\mathbf{\mathcal {A}}}_I)$ is an IOTA, which finally lets us conclude $\chi ^{\scriptstyle M}\!({\mathbf{\mathcal {A}}}_I)\ \mathbf{tioco}_M\ \chi ^{\scriptstyle M}\!({\mathbf{\mathcal {A}}}_S).$

$\boxed {\Longleftarrow }$ Assume that $\chi ^{\scriptstyle M}\!({\mathbf{\mathcal {A}}}_I)\ \mathbf{tioco}_M\ \chi ^{\scriptstyle M}\!({\mathbf{\mathcal {A}}}_S)$ with $M\in {\mathbb {R}}_{>0}$. This means that for all $\rho \in \textit{Sttraces}_M(\chi ^{\scriptstyle M}\!({\mathbf{\mathcal {A}}}_S))$ and for all $(d, o) \in \mathbf{{out}}_M(\chi ^{\scriptstyle M}\!({\mathbf{\mathcal {A}}}_I) \;\mathbf{after}_M\;\rho )$ we know that $(d, o) \in \mathbf{{out}}_M(\chi ^{\scriptstyle M}\!({\mathbf{\mathcal {A}}}_S) \;\mathbf{after}_M\;\rho )$.

To show that ${\mathbf{\mathcal {A}}}_I\ \mathbf{ioco}\ {\mathbf{\mathcal {A}}}_S$ according to Definition 5, we need to show that for all suspension traces of the specification $\sigma \in \textit{Straces}({\mathbf{\mathcal {A}}}_S)$ it holds that

$$\begin{aligned} \mathbf{{out}}\left( {\mathbf{\mathcal {A}}}_I\;{\mathbf {after}}\;\sigma \right) \subseteq \mathbf{{out}}\left( {\mathbf{\mathcal {A}}}_S\;{\mathbf {after}}\;\sigma \right) \end{aligned}$$

For that we will use induction.

*Base case.* Let $\sigma \in \textit{Straces}({\mathbf{\mathcal {A}}}_S)$. If $\sigma = \varepsilon$ with need to prove that for all $o\in \mathbf{{out}}({\mathbf{\mathcal {A}}}_I\;{\mathbf {after}}\;\varepsilon )$ it holds that $o\in \mathbf{{out}}({\mathbf{\mathcal {A}}}_S\;{\mathbf {after}}\;\varepsilon )$. With Theorem 1 and $\chi ^{\scriptstyle M}\!({\mathbf{\mathcal {A}}}_I)\ \mathbf{tioco}_M\ \chi ^{\scriptstyle M}\!({\mathbf{\mathcal {A}}}_S)$ this follows directly since $[\varepsilon ]\downarrow =\varepsilon$.

*Induction hypothesis.* Assume that for all timed traces *ρ * with length *n* it holds that if $o! \in \mathbf{{out}}({\mathbf{\mathcal {A}}}_I\;{\mathbf {after}}\;\sigma )$ then $o! \in \mathbf{{out}}({\mathbf{\mathcal {A}}}_S\;{\mathbf {after}}\;\sigma ).$ We continue to prove that for all timed traces $\sigma ' = a\cdot \sigma$ with length *n+1* it holds that if $o! \in \mathbf{{out}}({\mathbf{\mathcal {A}}}_I\;{\mathbf {after}}\;\sigma ')$ then $o! \in \mathbf{{out}}({\mathbf{\mathcal {A}}}_S\;{\mathbf {after}}\;\sigma ')$.

*Induction step.* Suppose there is an output such that $o! \in \mathbf{{out}}({\mathbf{\mathcal {A}}}_I\;{\mathbf {after}}\;\sigma ')$ and $o! \not \in \mathbf{{out}}({\mathbf{\mathcal {A}}}_S\;{\mathbf {after}}\;\sigma ').$ With Lemma 1 this means that exists a $d\in {\mathbb {R}}_{\ge 0}$ such that $(d, o) \in \mathbf{{out}}_M(\chi ^{\scriptstyle M}\!({\mathbf{\mathcal {A}}}_I) \;\mathbf{after}_M\;\rho ')$ and $(d, o) \not \in \mathbf{{out}}_M(\chi ^{\scriptstyle M}\!({\mathbf{\mathcal {A}}}_S) \;\mathbf{after}_M\;\rho ')$ where $[\rho ']\downarrow =\sigma '$. However, this cannot happen because $\chi ^{\scriptstyle M}\!({\mathbf{\mathcal {A}}}_I)\ \mathbf{tioco}_M\ \chi ^{\scriptstyle M}\!({\mathbf{\mathcal {A}}}_S)$. This implies for all output such that $o! \in \mathbf{{out}}({\mathbf{\mathcal {A}}}_I\;{\mathbf {after}}\;\sigma ')$ we know $o! \in \mathbf{{out}}({\mathbf{\mathcal {A}}}_S\;{\mathbf {after}}\;\sigma ')$, which concludes the induction and the proof.

*□*

Theorem 3 guarantees preservation of conformance, allowing practitioners to model only the system’s input–output behaviour. Quiescence and time-outs are added explicitly by the transformation described in Definition 12.

Technically, Theorem 3 works for any non-zero time-out *M*. In reality, practitioners should use the time-out time that works best in their domain.

## Testing with TAs

In this section we investigate the practical half of our testing theory. First we define test cases for LTSs and TAs, respectively. Our test cases are inspired from the literature in [1, 9] for the LTS case, and [4] for the TA case.

We present our core result that mirrors the practical side of **ioco** being preserved under the LTS-to-TA-transformation: Concretely, if an implementation passes every untimed test of its LTS specification, it will also pass every timed test of the lifted specification and conversely, any untimed test that shows non-conformance has a timed counterpart that reveals the same defect. Additionally, we show that $\chi ^{\scriptstyle M}\!$ commutes with test generation: applying $\chi ^{\scriptstyle M}\!$ before or after the standard ioco test-generation algorithm yields the same set of tests.

### Test Cases for LTS

We model test cases for an LTS ${\mathbf{\mathcal {A}}}$ as tree-shaped LTSs with the same inputs (representing what a tester may send) and same outputs (what the SUT may emit), and explicit *δ * actions in its transition relation. We note that test cases do not contain *τ * transitions, since test cases only model what should be observed during test execution, i.e. traces; by definition, *τ * is internal and cannot be observed.

The intuition of the format of test cases is that every state presents a choice for the tester: Either (1) stop—decide the verdict and end the test, (2) observe—wait to see whether the SUT produces an output or remains quiescent, or (3) stimulate—send an input to the SUT. We note here that tests may be generated according to this structure [1]). The last option, to send an input, comes with one caveat: while we are about to apply an input, the SUT is still allowed to “interrupt” us with an output. To handle races like this cleanly, the test tree always enables the entire output set alongside the chosen input. Notably, quiescence is *not* enabled in such a state, since we want to apply the input before the (as of yet time-agnostic) quiescence-timeout.

#### Definition 14

(*Test case for LTS*) A test case for an LTS ${\mathbf{\mathcal {A}}}_S$ is an LTS ${{\mathbf {t}}} =\langle S^{{\mathbf {t}}}, \textit{Act}^\delta , \rightarrow ^{{\mathbf {t}}}, s_0^{{\mathbf {t}}} \rangle$ s.t.:

- ${{\mathbf {t}}}$ uses the same action labels as ${\mathbf{\mathcal {A}}}_S$ plus *δ *
- ${{\mathbf {t}}}$ has only finite traces, is deterministic and has no cycles
- There are two special states **pass**, **fail**$\in S^{{\mathbf {t}}}$
- States **pass** and **fail** have no outgoing transitions: $\forall \ a\in \textit{Act}^\delta : {\mathbf {pass}} \mathop{\not\rightarrow}\limits^a \wedge \ {\mathbf {fail}} \mathop{\not\rightarrow}\limits^a$
- Every other state enables all outputs $\textit{Act}_O$, and either one input or *δ *, i.e. $\forall \ s\in S^{{\mathbf {t}}} \setminus \{{\mathbf {pass}},{\mathbf {fail}} \}:$
  $$ \begin{aligned} (|\mathbf{{in}}(s)|\!=\!0 \wedge \mathbf{{out}}(s)\!=\!\textit{Act}_O^\delta ) \\ \vee (\mathbf{{out}}(s)\!=\!\textit{Act}_O\wedge |\mathbf{{in}}(s)|\!=\!1) \end{aligned}$$
- Input-specifiedness: All traces of $${{\mathbf {t}}} $$ that end with an input are suspended traces of $${\mathbf{\mathcal {A}}}_S$$, i.e. $$ \forall \;\sigma \in (Act^{\delta } )^{*} $$$$ :\forall \;i? \in Act_{I} $$$$ :\sigma \cdot i? \in traces({\mathbf{t}}) $$$$ \Rightarrow \sigma \cdot i? \in Straces({\mathbf{\mathcal{A}}}_{S} ) $$
- Soundness: All traces of ${{\mathbf {t}}}$ leading to **pass**, are suspended traces of ${\mathbf{\mathcal {A}}}_S$
  $$\forall \ \sigma \in \textit{traces}({{\mathbf {t}}}): {{\mathbf {t}}} \xrightarrow {\sigma } {\mathbf {pass}} \Rightarrow \sigma \in \textit{Straces}({\mathbf{\mathcal {A}}}_S)$$
- Correctness: All traces of ${{\mathbf {t}}}$ that end with an output and lead to **fail**, are not suspended traces of ${\mathbf{\mathcal {A}}}_S$:
  $$\forall \ \sigma \in (\textit{Act}^\delta )^*:\forall \ o! \in \textit{Act}_O:\sigma \!\cdot \!o! \in \textit{traces}({{\mathbf {t}}}) \wedge {{\mathbf {t}}} \xrightarrow {\sigma \!\cdot \!o!}{\mathbf {fail}} \Rightarrow \sigma \!\cdot \!o! \not \in \textit{Straces}({\mathbf{\mathcal {A}}}_S).$$

Running a test case ${{\mathbf {t}}}$ against an implementation IOTS ${\mathbf{\mathcal {A}}}_I$ may yield different traces, due to the presence of nondeterministic choices in ${\mathbf{\mathcal {A}}}_I$. Then ${{\mathbf {t}}}$ fails on ${\mathbf{\mathcal {A}}}_I$ if at least one of the traces of ${\mathbf{\mathcal {A}}}_I$ leads to a fail-verdict in ${{\mathbf {t}}}$.

#### Definition 15

(*Test verdict*) Let ${{\mathbf {t}}}$ be a test case for an LTS ${\mathbf{\mathcal {A}}}_S$, and let ${\mathbf{\mathcal {A}}}_I$ be an implementation IOTS,

$$\begin{aligned} {\mathbf{\mathcal {A}}}_I\text { fails } {{\mathbf {t}}}\ \text { iff } \ \exists \ \sigma \in \textit{Straces}({\mathbf{\mathcal {A}}}_I) \cap \textit{traces}({{\mathbf {t}}}): {{\mathbf {t}}} \overset{\sigma }{\rightarrow }{\mathbf {fail}} \end{aligned}$$

Likewise, $${\mathbf{\mathcal {A}}}_I$$ passes $${{\mathbf {t}}} $$ iff $${\mathbf{\mathcal {A}}}_I$$$${{\mathbf {t}}} $$.

We note that we do not consider asynchronous inconsistencies in communication between the tester and the SUT, where input sending from the test case and output observation from the SUT may conflict with each other [9]. In this paper, we abstract from this and just deal with the traces that have been executed [1]. We could extend this in future work along the lines of [9]; our paper is then still valid, since the conflict resolution yields the trace that has actually been executed.

### Test Cases for TA

We model *timed* test cases for a TA as tree-shaped TAs that reuse the same inputs, outputs and explicit action *δ. * All intuitions from the LTS test case model carry over, but two timed-specific tweaks matter: (1) Time can elapse while we *observe*. In every node of the TA time may now elapse as long as the state invariant holds. Quiescence is detected when *no* output occurs during such a delay. The concrete quiescence-timeout bound is encoded as a guard on the transition. (2) Races now involve outputs *and* time. When we decide to *stimulate* the system, we may do so with some delay smaller than the quiescence delay. This makes the race between the SUT emitting output and us providing input explicit. We note that we restrict our timed test cases to *canonic* TAs of LTSs, because this excludes all timed automata with multiple clocks, or non-trivial invariants and guards.

#### Definition 16

(*Test case for TA*) A test case ${{\mathbf {t}}} _{\textit{TA}}$ for ${\mathscr {A}}_S$ is a ${{\mathbf {t}}} _{\textit{TA}} =\langle L^{{\mathbf {t}}}, \textit{Act}^\delta , \Phi ^{{\mathbf {t}}} _{L^{{\mathbf {t}}}}, {\mathcal {C}}^{{\mathbf {t}}}, \rightarrow ^{{\mathbf {t}}}, \ell ^{{\mathbf {t}}} _0\rangle$ such that:

- ${{\mathbf {t}}} _{\textit{TA}}$ is a canonic TA for a given ${\mathbf{\mathcal {A}}}_S$ and $M\in {\mathbb {R}}_{>0}$
- ${{\mathbf {t}}} _{\textit{TA}}$ uses the same action labels as ${\mathscr {A}}_S$ plus *δ *
- There are two special locations **pass**, **fail**$\in L^{{\mathbf {t}}}$
- Locations **pass** and **fail** have no outgoing transitions:

**Figure:**
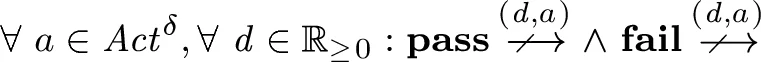
- ${{\mathbf {t}}} _{\textit{TA}}$ has only finite traces and has no cycles
- $${{\mathbf {t}}} _{\textit{TA}} $$ is deterministic, i.e.
  $$\forall \ a\in \textit{Act}^\delta , \forall \ {\textit{d}}\in {\mathbb {R}}_{\ge 0}, \forall \ \ell \in L^{{\mathbf {t}}}: |\ell \;\mathbf{after}_M\;(d,a)| \le 1$$
- Every location, except **pass** and **fail**, enables all outputs $\textit{Act}_O$, and either one input or *δ *, i.e. $\forall \ \ell \in L^{{\mathbf {t}}} \setminus \{{\mathbf {pass}},{\mathbf {fail}} \}$: $(|\mathbf{{in}}(\ell )|\!=\!0 \wedge \mathbf{{out}}(\ell )=\!\textit{Act}_O^\delta ) \vee (\mathbf{{out}}(\ell )\!=\!\textit{Act}_O\wedge |\mathbf{{in}}(\ell )|\!=\!1)$
- All locations except **pass** and **fail** have the invariant *c≤ M*, i.e. $\forall \ \ell \in L^{{\mathbf {t}}} \setminus \{{\mathbf {pass}},{\mathbf {fail}} \}: \Phi _L^{{\mathbf {t}}} (\ell ) = (c\le M)$
- All non-*δ * transitions have clock guard *c< M*, i.e. $\forall \ \langle \ell , a, \phi , \lambda , \ell '\rangle \in \ \rightarrow ^{{\mathbf {t}}}: a\ne \delta \implies \phi = (c< M)$
- All *δ * transitions have clock guard *c= M*, i.e. $\forall \ \langle \ell , a, \phi , \lambda , \ell '\rangle \in \ \rightarrow ^{{\mathbf {t}}}: a= \delta \implies \phi = (c= M)$
- Input-specifiedness: all traces of $${{\mathbf {t}}} _{\textit{TA}} $$ that end with an input are suspended traces of $${\mathscr {A}}_S$$, i.e. $$\forall \ \rho \in \textit{ttraces}({{\mathbf {t}}} _{\textit{TA}}), \forall \ i? \in$$$$\textit{Act}_I, \forall \ d \in {\mathbb {R}}_{\ge 0}: d \le M \wedge \rho \,\cdot \,(d,i?) \in$$$$\textit{ttraces}({{\mathbf {t}}} _{\textit{TA}}) \Rightarrow \rho \,\cdot \,(d,i?) \in$$$$\textit{Sttraces}_M({\mathscr {A}}_S)$$
- Soundness: All timed traces of $${{\mathbf {t}}} _{\textit{TA}} $$ leading to **pass**, are suspended timed traces of $${\mathscr {A}}_S$$$$\forall \ \rho \in \textit{ttraces}({{\mathbf {t}}} _{\textit{TA}}): {{\mathbf {t}}} _{\textit{TA}} \xrightarrow {\rho } {\mathbf {pass}} \Rightarrow \rho \in \textit{Sttraces}_M({\mathscr {A}}_S)$$
- Correctness: All timed traces of ${{\mathbf {t}}} _{\textit{TA}}$ that end with an output and lead to **fail**, are no suspended timed traces of ${\mathscr {A}}_S$:
  $$\forall \ \rho \!\cdot \!({\textit{d}},o!) \in \textit{ttraces}({{\mathbf {t}}} _{\textit{TA}}), \forall \ o! \in \textit{Act}_O, \forall \ {\textit{d}}\in {\mathbb {R}}_{\ge 0}: \\ d \le M \wedge {{\mathbf {t}}} _{\textit{TA}} \xrightarrow {\rho \!\cdot \!({\textit{d}},o!)}{\mathbf {fail}} \Rightarrow \rho \!\cdot \!({\textit{d}},o!) \not \in \textit{Sttraces}_M({\mathscr {A}}_S).$$

Similarly, running a test case ${{\mathbf {t}}}$ against an implementation IOTA ${\mathbf{\mathcal {A}}}_I$ may yield different traces, due to the presence of nondeterministic choices in ${\mathbf{\mathcal {A}}}_I$. Then ${{\mathbf {t}}}$ fails on ${\mathbf{\mathcal {A}}}_I$ if at least one of the traces of ${\mathbf{\mathcal {A}}}_I$ leads to a fail-verdict in ${{\mathbf {t}}}$.

#### Definition 17

(Timed test verdict) Let ${{\mathbf {t}}} _{\textit{TA}}$ be a test case for a TA ${\mathscr {A}}_S$, and let ${\mathscr {A}}_I$ be an implementation IOTA. We say:

$$\begin{aligned} {\mathscr {A}}_I\text { fails } {{\mathbf {t}}} _{\textit{TA}} \text {\ iff \ } \exists \ \rho \in \textit{Sttraces}_M({\mathscr {A}}_I)\cap \textit{ttraces}({{\mathbf {t}}} _{\textit{TA}}):{{\mathbf {t}}} _{\textit{TA}} \xrightarrow {\rho } {\mathbf {fail}} \end{aligned}$$

Likewise, $${\mathscr {A}}_I$$ passes $${{\mathbf {t}}} _{\textit{TA}} $$ iff $${\mathscr {A}}_I$$$${{\mathbf {t}}} _{\textit{TA}} $$.

#### Example 5

**Fig. 5:**
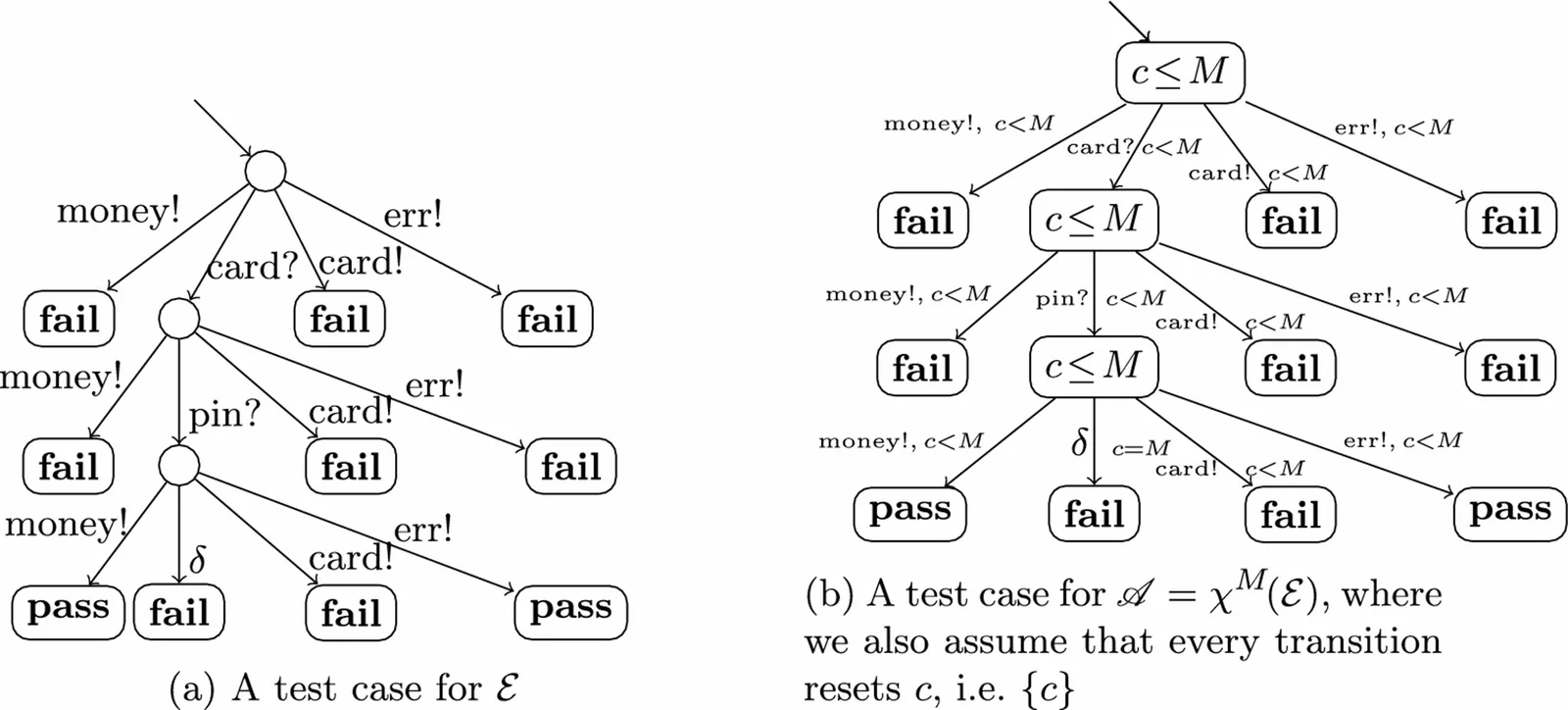
Corresponding test cases before and after $\chi ^{\scriptstyle M}\!$

For the LTS ${\mathcal {E}}$ in Fig. 4a, a corresponding test case is shown in Fig. 5baa. Similarly, Fig. 5ba(b) gives a test for the TA ${\mathscr {A}}$ of Fig. 4(b).

Every trace with at least one output that is not included in the specification is labelled ${\mathbf {fail}}$. This aligns with the concept of underspecifications in **ioco**. In practice, we generate test cases on-the-fly according to the specification model.

### Testing

The transformation in Definition 12 allows the specification activity to remain entirely in the untimed setting. A modeller provides a plain LTS that captures the functional behaviour of the intended system. From here *all* auxiliary machinery—adding quiescence and having a uniform time-out bound—is introduced automatically by $\chi ^{\scriptstyle M}\!$. Hence, the effort of dealing with time and quiescence is shifted from the modeller to the transformation.

Given Definitions 14 and 16, the next theorem establishes a one-to-one correspondence between the test suites obtained in the untimed and in the timed paradigm.

#### Theorem 4

(Test Correspondence) Let ${\mathbf{\mathcal {A}}}_S$ be an LTS and let ${{\mathbf {T}}} _{\textit{LTS}} ({\mathbf{\mathcal {A}}}_S)$ be the set of all its tests according to Definition 14. Similarly, let ${{\mathbf {T}}} _{\textit{TA}} (\chi ^{\scriptstyle M}\!({\mathbf{\mathcal {A}}}_S))$ be the set of all tests for $\chi ^{\scriptstyle M}\!({\mathbf{\mathcal {A}}}_S)$, which is a TA according to Definition 16. Then, for all $M\in {\mathbb {R}}_{>0}:$

$$\begin{aligned} \chi ^{\scriptstyle M}\!({{\mathbf {T}}} _{\textit{LTS}} ({\mathbf{\mathcal {A}}}_S)) = {{\mathbf {T}}} _{\textit{TA}} (\chi ^{\scriptstyle M}\!({\mathbf{\mathcal {A}}}_S)) \end{aligned}$$

#### Proof

Let $M\in {\mathbb {R}}_{>0}$. The proof is done in two steps:

$\boxed {\chi ^{\scriptstyle M}\!({{\mathbf {T}}} _{\textit{LTS}} ({\mathbf{\mathcal {A}}}_S)) \subseteq {{\mathbf {T}}} _{\textit{TA}} (\chi ^{\scriptstyle M}\!({\mathbf{\mathcal {A}}}_S))}$ Let ${{\mathbf {t}}} \in {{\mathbf {T}}} _{\textit{LTS}} ({\mathbf{\mathcal {A}}}_S)$ be an LTS test case for ${\mathbf{\mathcal {A}}}_S$ in correspondence with Definition 14. We must show that $\exists \ {{\mathbf {t}}} _{\textit{TA}} \in {{\mathbf {T}}} _{\textit{TA}} (\chi ^{\scriptstyle M}\!({\mathbf{\mathcal {A}}}_S)): \chi ^{\scriptstyle M}\!({{\mathbf {t}}})={{\mathbf {t}}} _{\textit{TA}}$ satisfies every clause of the TA-test definition (Definition 16). We go step-by-step below: 

*Structure*. The transformation $\chi ^{\scriptstyle M}\!$ performs a structure-preserving relabelling (cf. Definition 12, Lemma 1). It keeps the graph structure, i.e. nodes, quiescent states and transitions, adds one clock and decorates transitions and locations. Therefore, ${\mathbf {pass}}/{\mathbf {fail}}$ nodes, the tree shape and determinism are preserved.

*Invariants*. We show that for all locations $\ell \in L$ except ${\mathbf {pass}}$ and ${\mathbf {fail}}$ it holds that $\Phi _L(\ell )=\{c\le M\}$. By Definition 14${{\mathbf {t}}}$ either has exactly one outgoing input transition or exactly no outgoing input transition, i.e.

$$\begin{aligned} \forall \ \ell \in L: (|\mathbf{{in}}(\ell )|\!=\!0 \wedge \mathbf{{out}}(\ell )\!=\!\textit{Act}_O\cup \{\delta \}) \vee (\mathbf{{out}}(\ell )\!=\!\textit{Act}_O\wedge |\mathbf{{in}}(\ell )|\!=\!1) \end{aligned}$$

We point out once more that tests do not contain *τ *-actions. Irrespective of $\mathbf{{out}}(\ell )\!=\!\textit{Act}_O\cup \{\delta \}$ or $\mathbf{{out}}(\ell )\!=\!\textit{Act}_O$, according to the transformation (Definition 12) *ℓ * has the invariant *c≤ M*.

*Guards*. We show that the guard sets on transitions of ${{\mathbf {t}}} _{\textit{TA}}$ conform to the ones required of Definition 16 by making a distinction between inputs, non-*δ * outputs and *δ * outputs. According to  Definition 12:

- ... every input transition in ${{\mathbf {t}}} _{\textit{TA}}$ is of the form $\langle \ell ,i?,c<M,\{c\},\ell '\rangle$,
- ... every non-*δ * output transition in ${{\mathbf {t}}} _{\textit{TA}}$ is of the form $\langle \ell ,o!,c < M,\{c\},\ell '\rangle$
- ... every *δ * output transition in ${{\mathbf {t}}} _{\textit{TA}}$ is of the form $\langle \ell ,\delta ,c=M,\{c\},\ell '\rangle$

In all three cases this is exactly what is required in Definition 16. This means that all guard sets on transitions of ${{\mathbf {t}}} _{\textit{TA}}$ conform to Definition 16.

*Input-specifiedness, soundness, correctness*. Similar to the preservation in structure, $\chi ^{\scriptstyle M}\!$ neither adds nor deletes traces (cf. Lemma 1), so the three properties can be lifted unchanged.

All properties combined yield $\chi ^{\scriptstyle M}\!({{\mathbf {t}}})={{\mathbf {t}}} _{\textit{TA}}.$

$\boxed {\chi ^{\scriptstyle M}\!({{\mathbf {T}}} _{\textit{LTS}} ({\mathbf{\mathcal {A}}}_S)) \supseteq {{\mathbf {T}}} _{\textit{TA}} (\chi ^{\scriptstyle M}\!({\mathbf{\mathcal {A}}}_S))}$ Let ${{\mathbf {t}}} _{\textit{TA}} \in {{\mathbf {T}}} _{\textit{TA}} (\chi ^{\scriptstyle M}\!({\mathbf{\mathcal {A}}}_S)).$ We need to show that there is a test ${{\mathbf {t}}} \in {{\mathbf {T}}} _{\textit{LTS}} ({\mathbf{\mathcal {A}}}_S)$, such that ${{\mathbf {t}}} _{\textit{TA}} =\chi ^{\scriptstyle M}\!({{\mathbf {t}}})$. For that, we consider the projection of the test case ${{\mathbf {t}}} _{\textit{TA}}$ (cf. Definition 12) as a candidate, i.e. we consider $[{{\mathbf {t}}} _{\textit{TA}} ]\downarrow$ and show that it is the test case we are looking for. Clearly $[{{\mathbf {t}}} ]\downarrow =\langle L,\textit{Act}^\delta ,\rightarrow ',\ell _0\rangle$ where

$$\begin{aligned} \rightarrow ' = \{(\ell , a, \ell ')\in L\times \textit{Act}^\delta \times L\mid (\ell ,a,\phi ,\{c\},\ell '\in \rightarrow _{{{\mathbf {t}}} _{\textit{TA}}})\} \end{aligned}$$

The projection is trace preserving and does not add new behaviour (cf. Lemma 1). Specifically, *δ * labels are only explicitly added in quiescent states during the transformation, meaning that their LTS counterpart has a corresponding *δ * in the suspension traces. The same holds when we apply the transformation of Definition 12. Structurally, timed tests are conservative extensions of regular LTS test cases (cf. Definitions 14 and 16), hence we conclude ${{\mathbf {t}}} =[{{\mathbf {t}}} _{\textit{TA}} ]\downarrow \in {{\mathbf {T}}} _{\textit{LTS}}$. What remains to be shown is ${{\mathbf {t}}} _{\textit{TA}} =\chi ^{\scriptstyle M}\!({{\mathbf {t}}})$. For that we show that the location invariants and the transition guards are the same. 

*Invariants*. According to Definition 12 all locations have the clock invariant $\Phi =\{c\le M\}$. The same is true for timed test cases (cf. Definition 16).

*Guards*. Like before, the case distinction is between inputs, non-*δ * outputs and *δ * outputs. We observe that the transition guards in both Definitions 12 and 16 are

- *ϕ =(c< M)* for inputs
- *ϕ =(c< M)* for non-*δ * outputs and
- *ϕ =(c= M)* for *δ * outputs

This implies that the transition guards after transformation $\chi ^{\scriptstyle M}\!$ and those of timed test cases in ${{\mathbf {T}}} _{\textit{TA}}$ are the same.

Ultimately this yields ${{\mathbf {t}}} _{\textit{TA}} =\chi ^{\scriptstyle M}\!([{{\mathbf {t}}} _{\textit{TA}} ]\downarrow )$, and with it ${{\mathbf {t}}} _{\textit{TA}} \subseteq \chi ^{\scriptstyle M}\!({{\mathbf {T}}} _{\textit{LTS}} ({\mathbf{\mathcal {A}}}_S))$. *□ *

Theorem 4 shows that test derivation and the transformation $\chi ^{\scriptstyle M}\!$ commute: transforming a test after it is generated yields exactly the same result as generating the test after the specification has been transformed. This commutation property and Theorem 5 are the technical core of our presented work, because both guarantee that every verdict reached in the untimed paradigm is mirrored in the timed one. We therefore lift correctness properties from $\mathbf{ioco}$ to $\mathbf{tioco}_M$.

#### Theorem 5

Let $M\in {\mathbb {R}}_{>0}$, ${\mathbf{\mathcal {A}}}_I$ be an IOTS and ${\mathbf{\mathcal {A}}}_S$ be an LTS. Let ${{\mathbf {T}}} _{\textit{LTS}} ({\mathbf{\mathcal {A}}}_S)$ and ${{\mathbf {T}}} _{\textit{TA}} (\chi ^{\scriptstyle M}\!({\mathbf{\mathcal {A}}}_S))$ be the set of all annotated tests for ${\mathbf{\mathcal {A}}}_S$ and $\chi ^{\scriptstyle M}\!({\mathbf{\mathcal {A}}}_S)$, respectively. Then:

1. If $${\mathbf{\mathcal {A}}}_I\ \text {passes } {{\mathbf {T}}} _{\textit{LTS}} ({\mathbf{\mathcal {A}}}_S)$$, then $$\chi ^{\scriptstyle M}\!({\mathbf{\mathcal {A}}}_I)\ \text {passes } {{\mathbf {T}}} _{\textit{TA}} (\chi ^{\scriptstyle M}\!({\mathbf{\mathcal {A}}}_I))$$
2. If $$ {\mathbf{\mathcal {A}}}_I\ \text {fails } {{\mathbf {T}}} _{\textit{LTS}} ({\mathbf{\mathcal {A}}}_S)$$, then $$\chi ^{\scriptstyle M}\!({\mathbf{\mathcal {A}}}_I)\ \text {fails } {{\mathbf {T}}} _{\textit{TA}} (\chi ^{\scriptstyle M}\!({\mathbf{\mathcal {A}}}_I)).$$

#### Proof

Let ${\mathbf{\mathcal {A}}}_I,{\mathbf{\mathcal {A}}}_S,M,{{\mathbf {T}}} _{\textit{LTS}} ({\mathbf{\mathcal {A}}}_S)$ and ${{\mathbf {T}}} _{\textit{TA}} (\chi ^{\scriptstyle M}\!({\mathbf{\mathcal {A}}}_S))$ be as specified.

- $\boxed {1}$ The proof is via contraposition, i.e. we prove if $\chi ^{\scriptstyle M}\!({\mathbf{\mathcal {A}}}_I)\ \text {fails } {{\mathbf {T}}} _{\textit{TA}} (\chi ^{\scriptstyle M}\!({\mathbf{\mathcal {A}}}_S))$, then it follows that ${\mathbf{\mathcal {A}}}_I\ \text {fails } {{\mathbf {T}}} _{\textit{LTS}} ({\mathbf{\mathcal {A}}}_S)$. Thus, assume $\chi ^{\scriptstyle M}\!({\mathbf{\mathcal {A}}}_I)\ \text {fails } {{\mathbf {T}}} _{\textit{TA}} (\chi ^{\scriptstyle M}\!({\mathbf{\mathcal {A}}}_S))$. By Definition 17 this implies that there is a timed test case ${{\mathbf {t}}} _{\textit{TA}} \in {{\mathbf {T}}} _{\textit{TA}} (\chi ^{\scriptstyle M}\!({\mathbf{\mathcal {A}}}_S))$ such that there is a (suspended) timed trace that leads to a fail state, i.e.
  $$\begin{aligned} \exists \ \rho \in \textit{Sttraces}_M(\chi ^{\scriptstyle M}\!({\mathbf{\mathcal {A}}}_I))\cap \textit{ttraces}_M({{\mathbf {t}}} _{\textit{TA}}):{{\mathbf {t}}} _{\textit{TA}} \overset{\rho }{\longrightarrow } {\mathbf {fail}} \end{aligned}$$
  Note that tests do not have *τ * transitions with time nor without time. For this timed trace *ρ * let $\sigma =[\rho ]\downarrow$ be its projected trace (cf. Definition 13). From here we deduce two properties:
  1. Since $\rho \in \textit{Sttraces}_M(\chi ^{\scriptstyle M}\!({\mathbf{\mathcal {A}}}_I))$ we have $\sigma \in \textit{Straces}({\mathbf{\mathcal {A}}}_I)$ (cf. Lemma 1), and
  2. By the definitions of test cases (cf. Definition 14) and timed test cases (cf. Definition 16) we know that this trace leads to a fail state in the untimed test ${{\mathbf {t}}} =[{{\mathbf {t}}} _{\textit{TA}} ]\downarrow$, i.e. $\sigma \in \textit{traces}({{\mathbf {t}}}) \text { and } {{\mathbf {t}}} \overset{\sigma }{\longrightarrow }{\mathbf {fail}}.$
  Moreover, with Theorem 4, $[{{\mathbf {t}}} _{\textit{TA}} ]\downarrow \in {{\mathbf {T}}} _{\textit{LTS}}$. This means we found an untimed test case ${{\mathbf {t}}} \in {{\mathbf {T}}} _{\textit{LTS}} ({\mathbf{\mathcal {A}}}_S)$ that contains a (suspended) trace *σ * which leads ${\mathbf{\mathcal {A}}}_I$ to a fail state. By Definition 14 this means that ${\mathbf{\mathcal {A}}}_I\ \text {fails } {{\mathbf {T}}} _{\textit{LTS}}.$
- $$\boxed {2}$$ Assume $${\mathbf{\mathcal {A}}}_I\ \text {fails } {{\mathbf {T}}} _{\textit{LTS}} $$, then according to Definition 15 there is $${{\mathbf {t}}} \in {{\mathbf {T}}} _{\textit{LTS}} ({\mathbf{\mathcal {A}}}_S)$$ for which there is $$\sigma \in \textit{Straces}({\mathbf{\mathcal {A}}}_I)\cap$$$$\cap \textit{traces}({{\mathbf {t}}})$$ with $${{\mathbf {t}}} \overset{\sigma }{\longrightarrow }{\mathbf {fail}}. $$ With Theorem 4 we know that $$\chi ^{\scriptstyle M}\!({{\mathbf {t}}})\in {{\mathbf {T}}} _{\textit{TA}} (\chi ^{\scriptstyle M}\!({\mathbf{\mathcal {A}}}_S)).$$ Likewise, with Lemma 1 we know there is $$\rho \in \textit{Sttraces}_M(\chi ^{\scriptstyle M}\!({\mathbf{\mathcal {A}}}_I))\cap \textit{ttraces}(\chi ^{\scriptstyle M}\!({{\mathbf {t}}}))$$ such that $$[\rho ]\downarrow =\sigma. $$ Notably, with the definition of the transformation (cf. Definition 12) this means we found
  $$\begin{aligned} \rho \in \textit{Sttraces}_M(\chi ^{\scriptstyle M}\!({\mathbf{\mathcal {A}}}_I))\cap \textit{ttraces}({{\mathbf {t}}} _{\textit{TA}}):{{\mathbf {t}}} _{\textit{TA}} \overset{\rho }{\longrightarrow }{\mathbf {fail}} \end{aligned}$$
  With Definition 17 this means $\chi ^{\scriptstyle M}\!({\mathbf{\mathcal {A}}}_I)\ \text {fails } {{\mathbf {t}}} _{\textit{TA}}$ and with it $\chi ^{\scriptstyle M}\!({\mathbf{\mathcal {A}}}_I)$ fails ${{\mathbf {T}}} _{\textit{TA}} (\chi ^{\scriptstyle M}\!({\mathbf{\mathcal {A}}}_S))$.

*□ *

## *M*-Urgent Traces and Action Hiding

For constructing larger models, it is often useful to hide actions that represent only internal details, or details that are otherwise deemed uninteresting by a tester. However, action hiding may influence the delay visible for the following action, and hence the general bound *M* that we set as a time-out for quiescence. Since time may pass for a *τ *-transition to be taken, we need to increase bound *M*. Before addressing this, we first define the concept of *M*-urgent languages, where we just reason about all traces respecting the bound *M*, without looking at the structure of the automaton itself. We show that our transformation $\chi ^{\scriptstyle M}\!$ naturally yields TAs with a language (i.e. set of traces) that is *M*-urgent. In the next part, we show how action hiding relates to *M*-urgency.

### Definition 18

(*M*-*urgency*) Let $$M \in {\mathbb {R}}_{>0}$$.

- For any suspended timed trace $\rho = (d_1,a_1) \dots (d_n,a_n) \in \textit{Sttraces}({\mathscr {A}})$ we say that it is *ρ * is *M*-urgent if for all $1 \le i \le n$:
  - if $a_i \in \textit{Act}_O$ then $d_i < M$, and
  - if $$a_i = \delta $$ then $$d_i = M$$
- Let ${\mathscr {A}}$ be a TA. We define its language as ${\mathcal {L}}({\mathscr {A}}) = \textit{Sttraces}({\mathscr {A}})$
- A language *L* is *M*-urgent if all $\rho \in L$ are *M*-urgent.

### Theorem 6

Let ${\mathbf{\mathcal {A}}}$ be an LTS. Then ${\mathcal {L}}(\chi ^{\scriptstyle M}\!({\mathcal {A}}))$ is *M*-urgent.

### Proof

Let $\rho = (d1,a_1) \dots (d_n,a_n )\in \textit{Sttraces}(\chi ^{\scriptstyle M}\!({\mathcal {A}}))$. Then by the definition of the transformation (cf. Definition 12), we have that each $(d_i,a_i)$ corresponds to one of the following transitions of $\chi ^{\scriptstyle M}\!({\mathcal {A}})$:

1. $$(\ell , i?, c<M, \{c\},\ell ')$$
2. $$(\ell , o!, c<M, \{c\},\ell ')$$
3. $$(\ell , \delta , c=M, \{c\},\ell ')$$
4. $$(\ell , \tau ,\{\},\{\},\ell ')$$

Recall that $\textit{Sttraces}(\chi ^{\scriptstyle M}\!({\mathcal {A}}))$ contains only *observable* timed traces, i.e., timed sequences over $\textit{Act}$. In particular, internal *τ *-transitions are not part of timed traces *ρ *. Moreover, *τ *-transitions in $\chi ^{\scriptstyle M}\!({\mathcal {A}})$ do not reset the clock *c* and are subject to the invariant *c≤ M* (cf. Definition 12). Hence, *τ * cannot delay the occurrence of an *observable* action beyond time *M*. It then follows that:

- if $a_i = o!$ or $a_i = i?$, then $d_i < M$
- if $$a_i = \delta $$, then $$d_i = M$$.

This is satisfies the requirements of the definition of *M*-urgency (Definition 18). *□ *

When action hiding is applied on an LTS, the transitions of a particular action are replaced by a *τ * transition. This way, the action is not observable anymore. Hiding can be useful in practice, e.g. for purposes such as encapsulation, where one only keeps actions visible that are needed for interaction with the environment, while the hidden actions model internal actions of the system.

### Definition 19

Let ${\mathcal {A}}= \langle S, \textit{Act}_\tau , \rightarrow , s_0\rangle$ be an LTS, and $a\in \textit{Act}$. The action hiding of *a* in ${\mathbf{\mathcal {A}}}$ yields the LTS $\textit{Hide}_{a}({\mathcal {A}}) = \langle S,\textit{Act}_\tau \setminus \{a\},\rightarrow ',s_0\rangle$ where:

$$ \rightarrow ' = \{(s,a',s') \in \rightarrow \mid a' \ne a\} \cup \{(s,\tau ,s') \mid (s,a,s')\in \rightarrow \wedge \ a' = a\} $$

Hiding an action however may violate preservation of *M*-urgency. Suppose that ${\mathcal {L}}(\chi ^{\scriptstyle M}\!({\mathcal {A}}))$ is *M*-urgent, and that in particular, it has a suspended timed trace $\rho = (d_1,a_1) \dots (d_i,a) (d_{i+1},a_{i+1}) \dots (d_n,a_n)$. After hiding for action *a*, this trace may be observed as $\rho ' = (d_1,a_1) \dots (d_i + d_{i+1},a_{i+1}) \dots (d_n,a_n)$, since the *τ * is not observable, and thus not occurring in a suspended timed trace (Definition 9). If $d_i + d_{i+1} > M$ and $a{a_{i+1}} \in \textit{Act}_O^\delta$ is an output or *δ *, then trace *ρ '* is not *M*-urgent. Moreover, if $d_i + d_{i+1} > M$, intuitively, quiescence could have occurred after exactly duration *M* instead. This is solved by increasing *M* to a value that is truly an upper bound. The following theorem states that upper bound *(x +1) · M* is always sufficient for a system that has traces with at most *x* subsequent occurrences of hidden action *a*.

### Theorem 7

Let ${\mathcal {A}}= \langle S, \textit{Act}_\tau , \rightarrow , s_0\rangle$ be an LTS, $a\in \textit{Act}$ an action, and $M \in {\mathbb {R}}_{>0}$ so that ${\mathcal {L}}(\chi ^{\scriptstyle M}\!({\mathcal {A}}))$ is *M*-urgent. Let $x \in {\mathbb {N}}$. If for any $\rho \in \textit{Sttraces}(\chi ^{\scriptstyle M}\!({\mathcal {A}}))$, action *a* occurs at most *x* times subsequently in *ρ *, then ${\mathcal {L}}(\chi ^{\scriptstyle {(x+1)\cdot M}}\!(\textit{Hide}_{a}({\mathcal {A}})))$ is *(x+1)· M*-urgent.

### Proof

Let $\rho = (d_1,a_1) \dots (d_n,a_n) \in \textit{Sttraces}(\chi ^{\scriptstyle M}\!({\mathcal {A}}))$. If *n=1*, then hiding is trivial, as there is no accumulation of delays between observable actions; hence the *M*-urgency condition is preserved. Therefore assume *n>1* and suppose that for some $1 \le i \le j < n$ with *j-i ≤ x* we have for all $i \le k \le j$ that $a_k = a$. We have two cases:

- If $a_{j+1} = \delta$ then in $\chi ^{\scriptstyle {(x+1)\cdot M}}\!(\textit{Hide}_{a}({\mathcal {A}}))$, the trace will occur as $\rho '= (d_1,a_1) \dots ((x+1)\cdot M, \delta )\dots (d_n,a_n)$ by definition of $\chi ^{\scriptstyle {(x+1)\cdot M}}\!$. Because $M \le (x+1)\cdot M$ we have for *1 ≤ l < i* and *j < l ≤ n* that $d_l < (x+1)\cdot M$ if $a_l \in \textit{Act}$, and $d_l = (x+1)\cdot M$ if $a_l = \delta$. Consequently, *ρ '* is *(x+1)· M*-urgent.
- If $a_{j+1} \in \textit{Act}$ then in $\chi ^{\scriptstyle {(x+1)\cdot M}}\!(\textit{Hide}_{a}({\mathcal {A}}))$ this trace will occur as $\rho ' = (d_1,a_1) \dots (d_i+ \dots + d_j +d_{j+1},a_{j+1}) \dots (d_n,a_n).$ Since *ρ * is *M*-urgent and $a\in \textit{Act}$, we have that $d_k < M$ for all $i \le k \le j$. Hence, $d_i+ \dots + d_j +d_{j+1},a_{j+1} < (x+1)\cdot M$. Exactly as in the previous case, we have for *l < i* or *l > j* that $d_l < (x+1)\cdot M$ if $a_l \in \textit{Act}$, and $d_l = (x+1)\cdot M$ if $a_l = \delta$. Consequently, *ρ '* is *(x+1)· M*-urgent.

*□*

We note that in practice, upper bound *(x+1)· M* may be rather large, and possibly also not necessary to use, if it is never needed to wait that long. In particular, if a hidden output only needs at most *N < M* delay, or if a hidden input is always provided within a delay of *N < M*, the bound *x· N + M* can be used.

## Related Work

**Timed conformance.** The testing of real-time systems has long been a topic of research interest. Early work extended the theory of testing deterministic Mealy machines to timed input/output automata [10]. Later, [11] expand these results by considering a determinizable class of non-deterministic timed systems. From there, much of the theory evolved into LTS-based ioco frameworks [12]. This is because LTSs capture non-deterministic branching while maintaining an operational view of inputs and outputs. The best-known timed variations are tioco [2], rtioco [3], and the variant adopted here, $\mathbf{tioco}_M$ [4]. Also of note are dtioco for distributed systems and multi-traces [13] and live timed ioco (ltioco), which further distinguishes two flavours of quiescence and works directly on TA zone graphs [14]. Other work combines timed properties with probabilities [15, 16].

Timed testing remains a topic of interest, even beyond ioco and LTSs, e.g. [17] use timed FSMs, and [18] augment CSP with discrete time. The work of [19] brings forth decidability results for TAs with one clock–which relates to our systems after the transformation $\chi ^{\scriptstyle M}\!$.

**Quiescence and ioco.** Tretmans [12] introduced quiescence in his seminal work on ioco theory via suspension traces, which include *δ * to denote the absence of observable output. The concept was later refined, with Stokkink et al. treating *δ * as a first-class citizen and discussing well-formedness rules [1] and divergence [20]. Numerous ioco variants have been developed to suit different modelling contexts and application domains, including compositional ioco, i.e. uioco [21, 22], probabilistic ioco [23], symbolic ioco [24, 25], and modal ioco [26].

**Model transformations and tool support.** To operationalise conformance, design artefacts must first be transformed into testable models, then automated tools can execute the resulting test suites and provide verdicts. Two such related model-to-test transformations are provided by [27], who take the opposite route to ours and translate a TA into an untimed one to leverage the arsenal of available untimed testing techniques, and [28] who derive timed test cases directly from UML activity diagrams. Noteworthy tools of industrial maturity realise these theories, e.g. UPPAAL Tron [2] executes tioco test suites and RT-Tester [29] supports safety-critical certifications in the automotive and aviation industry.

## Conclusion

We provided a lightweight route for lifting untimed LTS models and test suites into the timed domain. Central to our approach is the canonic TA-ification operator $\chi ^{\scriptstyle M}\!$ (cf. Definition 12), which augments any LTS/IOTS with a single clock that models quiescence explicitly as a time-out at a user-chosen bound *M*. We provide proofs that $\chi ^{\scriptstyle M}\!$ preserves conformance from the classical **ioco** relation to $\mathbf{tioco}_M$ (cf.  Theorem 3), and, via a tight construction of test cases (cf. Definition 14 and Definition 16) Theorem 5 guarantees that every fail detectable in the untimed setting remains detectable once timing constraints are introduced via the transformation. Additionally, we showed that $\chi ^{\scriptstyle M}\!$ commutes with test generation: applying $\chi ^{\scriptstyle M}\!$ before or after the standard ioco test-generation algorithm yields the same set of tests. A key extension over our earlier work [5] is the explicit support for internal (*τ *) actions, which allows us to reason about action hiding and its effect on observable timed behaviour.

Overall, our work lowers the entry barrier for timed conformance testing: existing LTS specifications can be used as input for $\chi ^{\scriptstyle M}\!$, such that off-the-shelf tioco tooling can be used to generate and execute the timed tests of the resulting TA, in particular using UPPAAL-TRON [30]. This reflects how ioco-based testing with quiescence is often carried out in practice; our results provide the formal underpinning for this approach. While we explicitly support internal actions, an interesting direction for future work is to refine their interpretation further, for example by incorporating notions of divergence-as-quiescence as studied in [6]. A complementary research direction from the perspective of a practitioner is the automatic inference for optimal time-out bounds *M*, for example from observed traces or domain knowledge, which would reduce manual tuning even further.

## Data Availability

Not applicable.
